# Therapy Effect of the Stable Gastric Pentadecapeptide BPC 157 on Acute Pancreatitis as Vascular Failure-Induced Severe Peripheral and Central Syndrome in Rats

**DOI:** 10.3390/biomedicines10061299

**Published:** 2022-06-01

**Authors:** Ivan Maria Smoday, Igor Petrovic, Luka Kalogjera, Hrvoje Vranes, Helena Zizek, Ivan Krezic, Slaven Gojkovic, Ivan Skorak, Klaudija Hriberski, Ivan Brizic, Milovan Kubat, Sanja Strbe, Ivan Barisic, Marija Sola, Eva Lovric, Marin Lozic, Alenka Boban Blagaic, Anita Skrtic, Sven Seiwerth, Predrag Sikiric

**Affiliations:** 1 Department of Pharmacology, School of Medicine, University of Zagreb, 10000 Zagreb, Croatia; ivansmoday1@gmail.com (I.M.S.); lkalogjera9@gmail.com (L.K.); hrvoje.vranes@gmail.com (H.V.); zizekhelena@gmail.com (H.Z.); ivankrezic94@gmail.com (I.K.); slaven.gojkovic.007@gmail.com (S.G.); ivan.skorak@gmail.com (I.S.); klaudija.hriberski@gmail.com (K.H.); 2360999@gmail.com (I.B.); strbes@gmail.com (S.S.); inbarisic@gmail.com (I.B.); marijasola11@gmail.com (M.S.); abblagaic@mef.hr (A.B.B.); 2 Department of Surgery, School of Medicine, University of Zagreb, 10000 Zagreb, Croatia; igor.petrovic33@gmail.com; 3 Department of Forensic Medicine and Criminology, School of Medicne, 10000 Zagreb, Croatia; milovan.kubat@mef.hr; 4 Department of Pathology, School of Medicine, University of Zagreb, 10000 Zagreb, Croatia; eva.lovric@kb-merkur.hr (E.L.); sven.seiwerth@mef.hr (S.S.); 5 Department of Pediatric and Preventive Dentistry, School of Dental Medicine, University of Zagreb, 10000 Zagreb, Croatia; marin.lozic@yahoo.com

**Keywords:** gastric pentadecapeptide BPC 157, acute pancreatitis, vascular failure, severe peripheral and central syndrome, rats

## Abstract

We revealed the therapy effect of the stable gastric pentadecapeptide BPC 157 (10 μg/kg, 10 ng/kg ig or po) with specific activation of the collateral rescuing pathways, the azygos vein, on bile duct ligation in particular, and acute pancreatitis as local disturbances (i.e., improved gross and microscopy presentation, decreased amylase level). Additionally, we revealed the therapy’s effect on the acute pancreatitis as vascular failure and multiorgan failure, both peripherally and centrally following “occlusion-like” syndrome, major intoxication (alcohol, lithium), maintained severe intra-abdominal hypertension, and myocardial infarction, or occlusion syndrome, and major vessel occlusion. The application-sacrifice periods were ligation times of 0–30 min, 0–5 h, 0–24 h (cured periods, early regimen) and 4.30 h–5 h, 5 h–24 h (cured periods, delayed regimen). Otherwise, bile duct-ligated rats commonly presented intracranial (superior sagittal sinus), portal and caval hypertension and aortal hypotension, gross brain swelling, hemorrhage and lesions, heart dysfunction, lung lesions, liver and kidney failure, gastrointestinal lesions, and severe arterial and venous thrombosis, peripherally and centrally. Unless antagonized with the key effect of BPC 157 regimens, reversal of the inferior caval and superior mesenteric vein congestion and reversal of the failed azygos vein activated azygos vein-recruited direct delivery to rescue the inferior-superior caval vein pathway; these were all antecedent to acute pancreatitis major lesions (i.e., acinar, fat necrosis, hemorrhage). These lesions appeared in the later period, but were markedly attenuated/eliminated (i.e., hemorrhage) in BPC 157-treated rats. To summarize, while the innate vicious cycle may be peripheral (bile duct ligation), or central (rapidly developed brain disturbances), or peripheral and central, BPC 157 resolved acute pancreatitis and its adjacent syndrome.

## 1. Introduction

Stable gastric pentadecapeptide BPC 157 therapy (for review, see, i.e., [1,2,3,4,5,6,7]) might be important in the full extent of acute pancreatitis and general disturbances (for review, see, i.e., [8,9,10]), and might resolve rat acute pancreatitis and occluded bile duct in general vascular failure.

Therapeutically, BPC 157 exhibited particular recovering vascular effects when confronted with various intentionally induced severe vascular disturbances (i.e., major vessel occlusion [11,12,13,14,15,16,17,18,19,20,21], peripheral [11,12,13,14,15,16,17,18,19] and central [20,21], vessel compression (intra-abdominal hypertension) [22], endothelium-damaging agents [23,24,25] and procedures [26,27,28]). BPC 157 cured the bile duct ligation-induced acute pancreatitis and its concomitant gastrointestinal disturbances [29,30], and prevented and reversed bile duct ligation-induced liver cirrhosis [31] in rats. Considering the previous therapy effect in rats with vascular disturbances [14,15,16,20,22,23,24,25] as well as in bile duct-ligated rats [29,30,31], this novel study analyzing acute pancreatitis/vascular disturbances shows the particularities of bile duct ligation-induced vascular failure. As a cause-effect relation, vascular failure may occur even before full acute pancreatitis presentation with a severe occlusion-like syndrome, peripherally and centrally. Essentially (i.e., providing a common vascular disability point), this might correspond to the previously described syndrome commonly seen with an endothelium-damaging agent overdose, alcohol [25], lithium [24], myocardial infarction [23], and maintained intra-abdominal hypertension, grade III and IV [22], as well as corresponding to the described occlusion syndrome with major vessel occlusion, peripherally [11,12,13,14,15,16,17,18,19] or centrally [20]. Of note, all of these disturbances were consistently attenuated with the application of BPC 157 therapy and activation of the collateral pathways, relayed on the given injury [11,12,13,14,15,16,17,18,19,20,21,22,23,24,25]. As a particular therapy effect, the BPC 157 regimen was given as intragastric bolus, or continuously orally in drinking water, starting in the early or late advanced injury course (for review, see, i.e., [1,2,3,4,5,6,7]).

Being stable and native in human gastric juice for more than 24 h, an easy application (i.e., in ulcerative colitis trial, it was safe without adverse effects, and a lethal dose (LD1) was not achieved in toxicology studies) (for review, see i.e., [1,2,3,4,5,6,7]) may indicate the particular therapy potential of this stable gastric pentadecapeptide BPC 157 in acute pancreatitis. Furthermore, due to its particular vascular recovering potential (for review, see i.e., [6]), BPC 157 could be fully capable at resolving the complete noxious course, acute pancreatitis, general vascular failure and occlusion-like syndrome. As emphasized before, the particular resolution of the activation of the collateral pathways, relayed on the given injury, would mean that the stable gastric pentadecapeptide BPC 157 therapy may realize an effective upgrade of the cytoprotection maxim endothelium maintenance → epithelium maintenance (for review, see i.e., [6]) as a powerful cytoprotective agent. The recognition of the cytoprotection maxim endothelium maintenance→ epithelium maintenance is potentially an essential but unresolved active key of Robert’s and Szabo’s cytoprotection concept, although they were overwhelmingly focused on stomach cytoprotection [32,33,34]. Recently, in these terms (activation of the collateral pathways, relayed on the given injury) [11,12,13,14,15,16,17,18,19,20,21,22,23,24,25,26,27,28], we reviewed the greater pleiotropic cytoprotection potential of the stable gastric pentadecapeptide BPC 157 as a novel cytoprotection mediator largely involved in healing (for review, see i.e., [1,2,3,4,5,6]), inside and outside the gastrointestinal tract (i.e., BPC 157 counteracted leaky gut (for review, see i.e., [4]). Special areas of therapy could be the bile duct ligation-induced disturbances [29,30,31]. As a specific, novel, rapid therapy effect (for review, see i.e., [6]), competing with the Virchow triad, commonly present, vascular recovery was consistently noted when confronted with multiorgan failure due to severe vascular failure [6]. As counteraction was generally seen peripherally and centrally [14,15,16,20,22,23,24,25], it might also be possible in rats with an occluded bile duct. Intracranial (superior sagittal sinus) hypertension, portal and caval hypertension, aortal hypotension, progressing venous and arterial thrombosis peripherally and centrally, and ECG disturbances [14,15,16,20,22,23,24,25] were commonly counteracted. Congested (i.e., inferior caval vein and superior mesenteric vein) and failed (azygos vein) blood vessels, multiple organs lesions, heart, lung, liver, kidney, the gastrointestinal tract in particular, and muscle weakness, as well as brain lesions, eye lesions (increased intraocular pressure, retinal ischemia) and oxidative stress in tissues were markedly attenuated [14,15,16,19,20,21,22,23,24,25].

Moreover, the BPC 157-activated “bypassing key” might act through modulatory effects on the prostaglandins system (i.e., counteracting non-steroidal anti-inflammatory drugs’ (NSAIDs) toxicity (for review, see i.e., [35]) and leaky gut syndrome (for review, see, i.e., [4]), acting also as a free radical scavenger [4,36,37,38]) and through modulatory effects on the nitric oxide (NO) system (i.e., interaction with NO agents in various models and species) [39], and in particular through special BPC 157/NO system relations (for review, see, i.e., [39]). There is quite strong evidence that the combined maintained endothelium function (i.e., induced NO release of its own, opposed NO synthase (NOS) blocker N(gamma)-nitro-L-arginine methyl ester (L-NAME) hypertension, opposed NOS-substrate L-arginine-hypotension) [40,41] and maintained thrombocyte function (opposed L-NAME pro-thrombotic effect, opposed L-arginine anti-thrombotic effect) and coagulation pathways were not affected [42,43,44]. Namely, BPC 157 was shown to affect several molecular pathways [11,21,27,45,46,47,48,49,50,51]; in particular, controlling vasomotor tone and the activation of the Src-Caveolin-1-eNOS pathway [48,49].

Otherwise, without therapy, as might also be the case with the bile duct ligation-induced acute pancreatitis and antecedent and concomitant occlusion-like syndrome, these severe (or even deadly) disturbances progressed. With an innate inability to activate collateral pathways, direct vessel occlusion (the inferior caval vein syndrome [11], Pringle maneuver ischemia, reperfusion syndrome [12], Budd-Chiari syndrome [13], superior sagittal sinus occlusion syndrome [20], occluded superior mesenteric artery syndrome [15], occluded superior mesenteric vein syndrome [14], occluded superior mesenteric artery and vein syndrome [16], and episcleral veins cauterization-glaucoma syndrome [19]) was encountered. Likewise, the mentioned inability to activate the collateral pathways also resulted in harm to the failed vessels in alcohol intoxication syndrome [25], lithium intoxication syndrome [24], and isoprenaline-induced myocardial infarction syndrome [23] or maintained intra-abdominal hypertension, grade III and IV syndrome [22]. Of note, with the BPC 157 therapy and counteracting potential, there is a correspondingly high range of collateral blood vessels’ pathways that may be involved, i.e., the azygos vein (superior-inferior caval vein shunt to provide more direct blood flow delivery) [13,22,23,24], left ovarian vein [11], and inferior mesenteric vein in the porto-caval shunt [12]. More specifically, the inferior and superior anterior pancreaticoduodenal, pyloric vein in the superior mesenteric vein-portal vein shunt [14,16], and inferior mesenteric artery and inferior anterior pancreaticoduodenal artery [15,16], and centrally, (para)sagittal venous collateral circulation [20]. Thus, there may be an essential, rapid effect as a general defensive response, particularly matched for the acute pancreatitis, vascular failure and complete occlusion-like syndrome. Namely, BPC 157 therapy exhibited a particular recovering effect in the superior mesenteric vessels’ tributaries, and as already shown the azygos vein acting via direct blood delivery (i.e., avoiding both the lung and liver) [13,20,22,23,24], to reorganize blood flow and instantly attenuate the consequences of maintained occlusion-induced vascular failure and occlusion-like syndrome of major intoxication [13,20,22,23,24].

In conclusion, we suggest the stable gastric pentadecapeptide BPC 157 as a useful oral peptide therapy against acute pancreatitis and the extensive presentation of adverse effects such as the shared occlusion-like syndrome. The therapy effect might possibly follow the counteraction of the antecedent early noxious effects of occlusion-like syndrome, imminent vascular failure, and the activated rescuing pathway, which are so far less investigated. Thus, in the time line, in addition to the acute pancreatitis development (gross, microscopy, biochemistry), we investigated brain swelling and lesions, heart dysfunction, lung lesions, liver and kidney failure, gastrointestinal lesions, widespread arterial and venous thrombosis, venous congestion (i.e., inferior caval vein and superior mesenteric vein), and venous failure (azygos vein). Likewise, we assessed severe intracranial (superior sagittal sinus), portal and caval hypertension and aortal hypotension, and ECG recording.

## 2. Materials and Methods

### 2.1. Animals

Male Albino Wistar rats, 12 weeks old, 200 g body weight, bred in-house at the Animal Pharmacology Facility, School of Medicine, Zagreb, Croatia (registered with the Veterinary Directorate (Reg. No: HR-POK-007)), randomly assigned as 6 rats/group/interval were used in all experiments. Rats were acclimated for five days and randomly assigned to their respective treatment groups, housed in polycarbonate (PC) cages (identified with dates, number of study, group, dose, number and sex of each animal) at 20–24 °C, with a relative humidity of 40–70% and noise level of 60 dB, illumination 12 h per day (fluorescent lighting), a standard good laboratory practice (GLP) diet and fresh water ad libitum. Procedures were in line with the standard operating procedures (SOPs) of the Animal Pharmacology Facility, and the European Convention for the Protection of Vertebrate Animals used for Experimental and other Scientific Purposes (ETS 123). This study was approved by the local Ethics Committee. The ethical principles of the study complied with the European Directive 010/63/E, the Law on Amendments to the Animal Protection Act (Official Gazette 37/13), the Animal Protection Act (Official Gazette 135/06), the Ordinance on the protection of animals used for scientific purposes (Official Gazette 55/13), Federation of European Laboratory Animal Science Associations (FELASA) recommendations and the recommendations of the Ethics Committee of the School of Medicine, University of Zagreb. The experiments were assessed by observers blinded as to the treatment.

### 2.2. Drugs

Stable gastric pentadecapeptide BPC 157 (GEPPPGKPADDAGLV, molecular weight 1419; Diagen, Slovenia), a partial sequence of the human gastric juice protein BPC, which is freely soluble in water at pH 7.0 and in saline, was prepared as a peptide with 99% high-performance liquid chromatography (HPLC) purity, with 1-des-Gly peptide being the main impurity. The BPC 157 dose and application regimens (10 μg or 10 ng/kg given as an intragastric administration or continuously orally, in drinking water) were as described previously (i.e., without use of a carrier or peptidase inhibitor) (for review, see, i.e., [1,2,3,4,5,6]).

### 2.3. Experimental Protocol

In deeply anesthetized rats, intraperitoneal (ip)-injected 40 mg/kg thiopental (Rotexmedica, Germany) and 10 mg/kg diazepam (Apaurin; Krka, Slovenia) was injected to induce acute pancreatitis and concomitant vascular failure general syndrome. We induced ligation of the bile duct at its entry into the duodenum [34,35] with assessments at 30 min, 5 h and 24 h.

Rats received BPC 157 (10 μg or 10 ng/kg) therapy as an early regimen immediately upon bile duct ligation (i), or as a delayed regimen at time of the already advanced acute pancreatitis (ii). (i) As an early regimen, rats received BPC 157 or saline (5 mL/kg) as an intragastric administration immediately following ligation, for assessment at 30 min, 5 h and 24 h of ligation time. (ii) As a delayed regimen, the therapy (BPC 157 or saline (5 mL/kg)) was given as an intragastric administration at 4.30 h of ligation time for assessment at 5 h of ligation time, or BPC 157 as an oral administration, given continuously in drinking water from 5 h of ligation time until the sacrifice at 24 h of ligation time (0.16 μg/mL/rat, 0.16 ng/mL/rat) while controls received drinking water only (12 mL/day/rat).

After complete calvariectomy, recordings of brain swelling (before procedure, after ligation and therapy application, and before sacrifice) followed the procedure previously used in our vascular studies [14,15,16,20,22,23,24]. The calvariectomy procedure included, medially to the superior temporal lines and temporalis muscle attachments, 6 burr holes drilled in three horizontal lines (just basal from the posterior interocular line (two rostral burr holes), just rostral to the lambdoid suture (and transverse sinuses) on both sides (two basal burr holes) and in line between the basal and rostral burr holes (two middle burr holes)).

Rats were laparatomized again before sacrifice for the corresponding presentation of the peripheral vessels (azygos vein, superior mesenteric vein, portal vein, inferior caval vein) and corresponding organ lesions (i.e., acute pancreatitis, stomach lesion). Recording was performed with a camera attached to a VMS-004 Discovery Deluxe USB microscope (Veho, Dayton, OH, USA) at the end of the experiment and assessed as before [12,13,14,15,16,20,22,23,24].

### 2.4. Superior Sagittal Sinus, Portal, Superior Mesenteric and Caval Vein and Abdominal Aorta Pressure Recording

Recordings followed the procedure, described in detail in our previous vascular studies [14,15,16,20,22,23,24], involving deeply anesthetized rats, a cannula (BD Neoflon™ Cannula, Eysins, Switzerland) connected to a pressure transducer (78534C MONITOR/TERMINAL; Hewlett Packard, Palo Alto, CA, USA), inserted into the portal vein, inferior caval vein and superior sagittal sinus, as well as the abdominal aorta at the level of the bifurcation at 15 min, 30 min, 60 min, or 120 min ACS-time. A single burr hole in the rostral part of the sagittal suture made above the superior sagittal sinus was made for superior sagittal sinus pressure recording, and the superior sagittal sinus anterior part was cannulated using a Braun intravenous cannula; then, after laparotomy, pressure recordings in the portal vein, inferior vena cava, and abdominal aorta were performed.

Accordingly [12,13,14,15,16,20,22,23,24], superior sagittal sinus pressure of −24 to −27 mmHg, portal pressure of 3–5 mmHg, similar to that of the inferior vena cava although with values at least 1 mmHg higher in the portal vein, and abdominal aorta blood pressure values of 100–120 mmHg at the level of the bifurcation were considered as normal in healthy rats.

### 2.5. ECG Recording

ECGs were recorded continuously in deeply anesthetized rats for all three main leads by positioning stainless steel electrodes on all four limbs using an ECG monitor with a 2090 programmer (Medtronic, Minneapolis, MN, USA) connected to a Waverunner LT342 digital oscilloscope (LeCroy, Chestnut Ridge, NY, USA) before procedure, after ligation and therapy application, and before sacrifice. This arrangement enabled precise recordings, measurements, and analysis of ECG parameters [14,15,16,20,22,23,24].

### 2.6. Thrombus Assessment

Following sacrifice, the superior sagittal sinus, and peripherally, the portal vein, inferior caval vein, superior mesenteric artery, and abdominal aorta were removed from the rats, and the clots were weighed [12,13,14,15,16,20,22,23,24].

### 2.7. Brain Volume and Vessel Volume Presentation

The procedure from our previous vascular studies [14,15,16,20,22,23,24] was applied. Brain volume and vessel volume and heart volume and pancreas volume were proportional to the change in the brain, vessel, heart or pancreas surface area. The presentation of the brain and peripheral vessels (superior mesenteric vein, inferior caval vein and azygos vein) was recorded in deeply anaesthetized rats, with a camera attached to a VMS-004 Discovery Deluxe USB microscope (Veho, Dayton, OH, USA) [14,15,16,17,18,19,20,21,26,27,28]. The border of the brain (or veins, heart or pancreas) in the image was marked using ImageJ software and then the surface area of the brain (or veins, heart or pancreas) was measured. This was conducted with brain (or vein) images for healthy rats, and then for both the control (saline) group and the treated (BPC 157) group of rats at the same intervals after the application and at the time of sacrifice. The arithmetic mean of the surface areas was calculated for both groups. Then, the ratio of these two areas was calculated as ($\frac{A_{con}}{A_{bpc}}$), where Acon is the arithmetic mean brain (or veins) area of the control group and Abpc is the arithmetic mean brain (or veins, heart or pancreas) area of the treated group. Starting from the square cube law, Equations (1) and (2), an equation for the change in brain (or veins, heart or pancreas) volume proportional to the change in brain surface area (6) was derived. In expressions (1)–(5), *l* is defined as any arbitrary one-dimensional length of the brain (for example, rostro-caudal length of the brain), used only for defining the one-dimensional proportion (l2/l1) between two observed brains (or veins) and as an inter-factor (thus, not measured (6)) for deriving the final expression (6). The procedure was as follows:

square-cube law:

(1) $${\;A}_{2}=A_{1}\times {\left(\frac{l_{2}}{l_{1}}\right)}^{2}$$

square-cube law:

(2) $$V_{2}=V_{1}\times {\left(\frac{l_{2}}{l_{1}}\right)}^{3}$$

from (1), after dividing both sides by A1:

(3) $$\frac{A_{2}}{A_{1}}={\left(\frac{l_{2}}{l_{1}}\right)}^{2}$$

from (3), after taking the square root of both sides:

(4) $$\frac{l_{2}}{l_{1}}=\sqrt{\frac{A_{2}}{A_{1}}}$$

from (2), after dividing both sides by V1:

(5) $$\frac{V_{2}}{V_{1}}={\left(\frac{l_{2}}{l_{1}}\right)}^{3}$$

after incorporating expression (4) into Equation (5):

(6) $$\frac{V_{2}}{V_{1}}={\left(\sqrt{\frac{A_{2}}{A_{1}}}\;\right)}^{3}$$

### 2.8. Gross Assessment of Gastrointestinal and Pancreas Lesions

For recording, we used a camera attached to a VMS-004 Discovery Deluxe USB microscope (Veho, Dayton, OH, USA). As described before, gross lesions in the gastrointestinal tract and in the stomach (sum of the longest diameters, mm) were assessed in deeply anaesthetized rats, laparatomized before sacrifice [12,13,14,15,16,20,22,23,24]. The acute pancreatitis damage was assessed using the same macroscopic system as previously [29,30] (score 0: pancreas without changes; score 1: pancreas with edema only; score 2: separate hemorrhagic zones and/or foci of necrosis, largest diameters < 1 mm; score 3: separate hemorrhagic zones and/or foci of necrosis, largest diameters > 3 mm; score 4: confluent hemorrhagic zones and/or foci of necrosis, largest diameters > 6 mm; score 5: diffuse hemorrhagic zones and/or necrosis in whole pancreas).

### 2.9. Microscopy

As described in the previous studies [12,13,14,15,16,20,22,23,24], evaluation was made by light microscopy using an Olympus 71 digital camera and an Olympus BX51 microscope (OLYMPUS Europa SE & CO. KG). Digital images were saved as uncompressed 24-bit RGB TIFF files using the software program AnalySIS (Olympus Soft Imaging System GmbH, Munster, Germany). Representative tissue specimens (i.e., the brain, liver, kidney, pancreas, stomach, small and large intestine, lungs, and heart taken at the end of the experiment, fixed in 10% neutral buffered formalin (pH 7.4) at room temperature for 24 h) were embedded in paraffin, sectioned at 4 μm, and stained with hemalaun and eosin (H&E).

#### 2.9.1. Brain Histology

As described in the previous studies [12,13,14,15,16,20,22,23,24], the brain was dissected according to NTP-7, at Level 3 and 6 with neuroanatomic subsites presented in certain brain sections using coronal sections with three mandatory sections. We used a semiquantitative neuropathological scoring system, and the sum of the analyzed affected areas (0–4) (i) and karyopyknotic cells in the brain areas (0–4) (ii) made (i) + (ii) a combined score (0–8) as follows. (i) Specific affected brain areas (0–4) (score 0 indicates no histopathologic change), cerebral (NTP-7, Level 3), cerebellar cortex (NTP-7, Level 6), hippocampus, thalamus, and hypothalamus (NTP-7, Level 3) were assessed as follows: small, patchy, complete or incomplete infarcts (≤10% of area affected) (score 1); partly confluent or incomplete infarcts (20–30% of area affected) (score 2); large confluent complete infarcts (40–60% of area affected) (score 3); total disintegration of the tissue in the cortex; large complete infarcts (>75% of area affected) in the hypothalamus, thalamus, and hippocampus (score 4). (ii) Karyopyknotic cells in the affected brain areas (0–4) (score 0 indicates no change), cerebral (NTP-7, Level 3), cerebellar cortex (NTP-7, Level 6), hippocampus, thalamus, and hypothalamus (NTP-7, Level 3) were analyzed as follows: a few karyopyknotic neuronal cells (≤20%) (score 1); patchy areas of karyopyknotic cells (50%) (score 2); more extensive karyopyknotic areas (75%) (score 3); complete infarction (100%) (score 4).

We also assessed the neuronal pathological changes in acquired digital images saved as uncompressed 24-bit RGB TIFF files in the software program AnalySIS (Olympus Soft Imaging System GmbH, Munster, Germany) by performing quantitative analysis of the neuronal damage in the karyopyknotic areas. The neurons of the cortical cerebral region, cerebellar region, hippocampus, and hypothalamus were counted in 10 different high powered fields (HPF, 400×) and 3 to 5 serial sections of each sample were used for the count, as described in https://www.ncbi.nlm.nih.gov/pmc/articles/PMC5303860/ (accessed on 25 May 2022). The field size was 0.24 μm^2^.

We used four criteria for estimation of the edema: a pale myelin, sieve-like appearance of myelinated areas, dilation of perivascular and pericellular spaces and vacuolar appearance of the neuropil of gray matter. Edema was graded as heavy, moderate, slight or no edema (score 0–3). https://www.sciencedirect.com/science/article/pii/S0379073821001286 (accessed on 25 May 2022).

We estimated hemorrhage as a percentage of the affected brain area. Intraventricular hemorrhage was noted as present or absent.

#### 2.9.2. Lung Histology

The same scoring system as in the previous studies [12,13,14,15,16,20,22,23,24] was used to grade the degree of lung injury in lung tissue analysis. Each of the features (i.e., focal thickening of the alveolar membranes, congestion, pulmonary edema, intra-alveolar hemorrhage, interstitial neutrophil infiltration, and intra-alveolar neutrophil infiltration) was scored (0–3) as absent (0) or present to a mild (1), moderate (2), or severe (3) degree, and a final histology score was determined.

#### 2.9.3. Renal, Liver, and Heart Histology

The same scoring system as in the previous studies [12,13,14,15,16,20,22,23,24] was used to grade renal (i.e., the degeneration of Bowman’s space and glomeruli, degeneration of the proximal and distal tubules, vascular congestion, and interstitial edema), liver (i.e., vacuolization of hepatocytes and pyknotic hepatocyte nuclei, activation of Kupffer cells, and enlargement of sinusoids) and heart (i.e., dilatation and congestion of blood vessels within the myocardium and coronary arteries) histology. Each specimen was scored using a scale ranging from 0–3 (0: none, 1: mild, 2: moderate, and 3: severe) for each criterion, and a final histology score was determined (0: none, 1: mild, 2: moderate, and 3: severe).

#### 2.9.4. Gastrointestinal Histology

As in previous studies [12,13,14,15,16,20,22,23,24], we used a histologic scoring scale adapted from Chui and coworkers [52] for the intestinal tissue damage, scoring 0–5 (normal to severe) in three categories (mucosal injury, inflammation, and hyperemia/hemorrhage) for a total score of 0 to 15, as described by Lane and coworkers [53]. Illustratively, the assessment included the morphologic features of mucosal injury (i.e., different grades of epithelial lifting, villi denudation, and necrosis), inflammation (i.e., focal to diffuse, according to lamina propria infiltration or subendothelial infiltration), and hyperemia/hemorrhage (i.e., focal to diffuse, according to lamina propria or subendothelial localization).

#### 2.9.5. Pancreas Histology

We performed one longitudinal section through the whole pancreas. Six different variables determining severity of injury were assessed for the total surface and scored and carried out as described [54], and as follows:

Edema: 0 = absent; 0.5 = focal expansion of interlobar septe; 1 = diffuse expansion of interlobar septe; 1.5 = same as 1 + focal expansion of interlobular septe; 2 = same as 1 + diffuse expansion of interlobular septae; 2.5 = same as 2 + focal expansion of interacinar septe; 3 = same as 2 + diffuse expansion of interacinar septae; 3.5 = same as 3 + focal expansion of intercellular spaces; 4 = same as 3 + diffuse expansion of intercellular spaces.

Acinar necrosis; 0 = absent; 0.5 = focal occurrence of 1–4 necrotic cells/HPF; 1 = diffuse occurrence of 1–4 necrotic cells/HPF; 1.5 = same as 1 + focal occurrence of 5–10 necrotic cells/HPF; 2 = diffuse occurrence of 5–10 necrotic cells/HPF; 2.5 = same as 2 + focal occurrence of 11–16 necrotic cells/HPF; 3 = diffuse occurrence of 11–16 necrotic cells/HPF (foci of confluent necrosis); 3.5 = same as 3 + focal occurrence of >16 necrotic cells/HPF; 4 ≥ 16 necrotic cells/HPF (extensive confluent necrosis).

Hemorrhage and fat necrosis: 0 = absent; 0.5 = 1 focus; 1 = 2 foci; 1.5 = 3 foci; 2 = 4 foci; 2.5 = 5 foci; 3 = 6 foci; 3.5 = 7 foci; 4 = 8 or more foci.

The scoring criteria for hemorrhage were presented separately from the fat necrosis scoring criteria due to the morphological differences referring to hemorrhage between control and treated animals.

Inflammation and perivascular infiltrate: 0 = 0–1 intralobular or perivascular leukocytes/HPF; 0.5 = 2–5 intralobular or perivascular leukocytes/HPF; 1 = 6–10 intralobular or perivascular leukocytes/HPF; 1.5 = 11–15 intralobular or perivascular leukocytes/HPF; 2 = 16–20 intralobular or perivascular leukocytes/HPF; 2.5 = 21–25 intralobular or perivascular leukocytes/HPF; 3 = 26–30 intralobular or perivascular leukocytes/HPF; 3.5 = more than 30 leukocytes/HPF or focal microabscesses; 4 = more than 35 leukocytes/HPF or confluent microabscesses.

### 2.10. Analytical Method

Serum amylase, aspartate transaminase (AST), alanine transaminase (ALT) (U/L), total bilirubin, direct and indirect (μmol/L), creatinine (μmol/L), blood urea (mmol/L) and platelet count from the whole blood were measured according to the standard technique as described before [11,29,30,31,42,43]. While serum amylase was determined in all groups in all intervals, AST, ALT, total bilirubin, direct and indirect, creatinine, urea and platelet count was assessed at 0–24 h of the application-sacrifice period.

### 2.11. Immunohistochemistry

Deparaffinized placental tissue sections (4 μm thickness) were mounted on glass slides (Agilent Dako, Carpinteria, CA, USA). The slides were conjugated with primary antibodies for 30 min at room temperature, followed by antibody detection using Dako REAL Envision detection system (Agilent Dako, Carpinteria, CA, USA). EnVision FLEX Target Retrieval Solution, High pH (50×) (Dako Omnis) pH = 9 (Agilent Technologies, Inc., Santa Clara, CA, USA) was used for antigen retrieval according to the manufacturer’s instructions. Primary antibodies were mouse monoclonal anti-human CD20 (dilution 1:200, Cat No. IR604, Agilent Technologies, Inc., USA), rabbit monoclonal anti-human CD3 (dilution 1:200, Cat No. IR503, Agilent Technologies, Inc., USA), mouse monoclonal anti-human CD68 (PG-M1) (dilution 1:100, Cat No. IS613, Agilent Technologies, Inc., Santa Clara, CA, USA), mouse monoclonal anti-human CD163 (dilution 1:40, Cat No. CMC 163M-14, Cell Marque TM, Merck KGaA, Darmstadt, Germany). Negative controls were processed for all assays and were made in the same manner except the incubation step with primary antibody, which was omitted.

#### Quantitative Analysis of CD20, CD3, CD68 (PG-M1) and CD163 Protein Expression

The expression of CD20, CD3, CD68 (PG-M1) and CD163 proteins in pancreatic tissue was independently assessed by two board-certified pathologists (A.S., E.L.). The score was obtained as the number of positive cells per one power field (magnification 200×). All the discordant results were resolved at the double-headed microscope evaluation. Immunohistochemically stained slides were analyzed using an Olympus 71 digital camera and an Olympus BX51 microscope. Complete pancreatic tissue section was scanned at 100× magnification to assess the area showing the densest area with positively stained cells, the ‘hot spot’. Assessing the 100× magnification “hot spot”, consecutive use of magnification 200× in the same area was used until the densest area with positive cells was included within the 200× field. The “hot spots” at 200× magnification were eligible for analysis.

### 2.12. Statistical Analysis

Statistical analysis was performed by parametric one-way analysis of variance (ANOVA), with the Newman–Keuls post hoc test or the non-parametric Kruskal–Wallis test, and subsequently the Mann–Whitney U test to compare groups. Values are presented as the mean ± standard deviation (SD) and as the minimum/median/maximum. To compare the frequency difference between groups, the chi-squared test or Fischer’s exact test was used. *p* < 0.05 was considered statistically significant.

## 3. Results

We revealed that a permanently occluded bile duct at its entry into the duodenum inducing acute pancreatitis, also provoked vascular failure and a perilous syndrome occurring peripherally and centrally. Commonly, in all acute pancreatitis periods (i.e., 0–30 min, 0–5 h, 0–24 h, 4.30 h–5 h, 5 h–24 h), there was a highly noxious syndrome, similar to those previously described after major vessel occlusion (occlusion syndromes) or along with endothelium-damaging agent application, myocardial infarction, or intra-abdominal hypertension (“occlusion-like” syndromes) [14,15,16,20,22,23,24,25].

The stable gastric pentadecapeptide BPC 157 therapy application might have a prophylactic and curative effect on both acute pancreatitis (i.e., gross presentation, microscopy, serum amylase) and adjacent adverse syndrome (i.e., attenuated/counteracted intracranial (superior sagittal sinus) hypertension and aortal hypotension, major ECG disturbances, progressing arterial and vein thrombosis, lesions in the brain, heart, lungs, liver, kidneys, and gastrointestinal tract). The BPC 157 therapy effect was comparable to the previous BPC 157 therapy of the mentioned occlusion and occlusion-like syndromes [14,15,16,20,22,23,24,25]. For this effect, the common key finding may be the prompt and sustained activation of the azygos vein in the BPC 157-treated bile duct-ligated rats, the direct blood delivery from the inferior caval vein to the superior caval vein instantly breaking the vicious cycle.

### 3.1. A Perilous Syndrome Occurred Peripherally and Centrally

#### 3.1.1. Blood Pressure Disturbances

**Figure 1:**
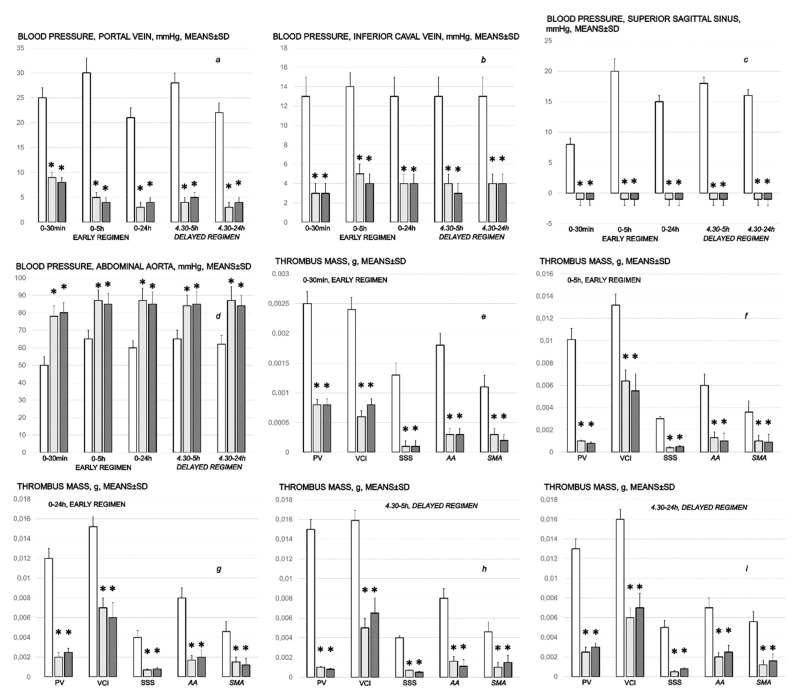
Blood pressure assessment, portal (**a**), caval (**b**) and intracranial (superior sagittal sinus) (**c**) hypertension, and aortal hypotension (**d**) in mmHg at the end of the ligation time period of 30 min, 5 h, and 24 h. Timeline of the thrombosis development in portal vein (PV), inferior caval vein (ICV), superior sagittal sinus (SSS), abdominal aorta (AA) and superior mesenteric artery (SMA) at the end of the ligation time period of 30 min, 5 h, and 24 h, as an early regimen at 0–30 min (**e**), 0–5 h (**f**), and 0–24 h (**g**) application-sacrifice period, and as delayed regimen at 4.30 h–5 h (**h**) and 5 h–24 h (**i**) application-sacrifice period. Therapy included BPC 157 (10 μg/kg (light gray bars) or 10 ng/kg (dark gray bars)) or saline (5 mL/kg) (white bars) as an intragastric administration either immediately following ligation, or as an early regimen, for assessment at 30 min, 5 h and 24 h-ligation time. As a delayed regimen, the therapy was given as an intragastric administration at 4.30 h ligation time for assessment at 5 h ligation time, or as oral administration, continuously in drinking water from 5 h ligation time until sacrifice at 24 h ligation time. Means ± SD, * *p* ˂ 0.05, at least, vs. control.

Perceived as a cause-effect relation that should be essentially affected by the therapy application, BPC 157 rapidly reduced blood pressure disturbances that were induced by bile duct ligation, which was quite severe peripherally (portal and caval hypertension, aortal hypotension) as well as centrally (superior sagittal sinus hypertension) (Figure 1). With a rapid and then sustained effect, the portal and caval hypertension and intracranial (superior sagittal sinus) hypertension as well as the aortal hypotension were eliminated, or at least markedly attenuated, with BPC 157 application.

#### 3.1.2. Thrombosis

Likewise, the fact that BPC 157 reduced thrombosis, both peripherally and centrally (Figure 1), may indicate the effective cause-effect course of the therapy. Without therapy, from the very beginning, as noted already at 30 min of ligation time, thrombosis progressed along with ligation time, peripherally in veins (i.e., portal vein and inferior caval vein) as well as in arteries (i.e., superior mesenteric artery, and abdominal aorta) and centrally (i.e., superior sagittal sinus).

#### 3.1.3. Collateral Pathways, Blood Vessels, and Brain Gross Presentation

**Figure 2:**
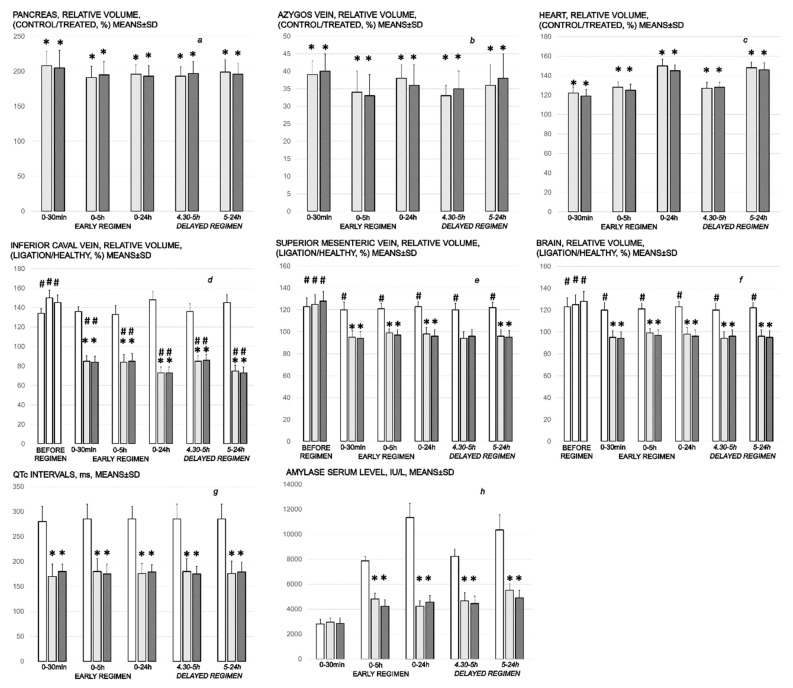
Timeline of relative volume (**a**–**f**) of the QTc intervals, ms (**g**), and serum amylase level, IU/L (**h**). Presentation of the relative volume of the pancreas (**a**), azygos vein (**a**), heart (**c**), inferior caval vein (**d**), superior mesenteric vein (**e**), and brain (**f**) to illustrate the effect on pancreas swelling, brain swelling, heart dilatation, superior mesenteric vein and inferior vein congestion, azygos vein failure, and counteraction as control/treated % or ligation/healthy % at the end of the ligation time period of 30 min, 5 h, and 24 h, as an early regimen at 0–30 min (**e**), 0–5 h (**f**), and 0–24 h (**g**) of application-sacrifice period, and as delayed regimen at 4.30 h–5 h (**h**) and 5 h–24 h application-sacrifice period. Therapy included BPC 157 (10 μg/kg (light gray bars) or 10 ng/kg (dark gray bars)) or saline (5 mL/kg) (white bars) as an intragastric administration either immediately following ligation or as an early regimen, for assessment at 30 min, 5 h and 24 h ligation time. As a delayed regimen, the therapy was given as an intragastric administration at 4.30 h ligation time for assessment at 5 h ligation time, or as oral administration, continuously in drinking water from 5 h ligation time until sacrifice at 24 h ligation time. Means ± SD, * *p* ˂ 0.05, at least, vs. control, # *p* ˂ 0.05, at least, vs. healthy.

**Figure 3:**
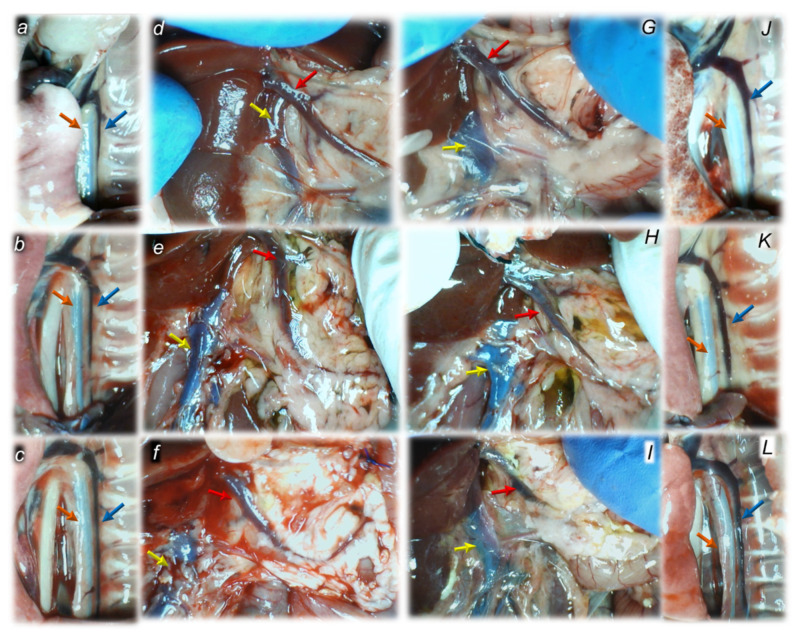
Regular timeline (30 min (**a**,**d**), 5 h (**b**,**e**) and 24 h (**c**,**f**) ligation time period) of disturbed vessels’ presentation, failed azygos vein (blue arrows) and thoracic aorta (orange arrows) (**a**–**c**), and congested superior mesenteric vein (red arrows) and inferior caval vein (yellow arrows) (**d**–**f**) in control rats with ligated bile duct. Upon BPC 157 therapy and recruitment of the defensive pathways, congestion of the superior mesenteric vein and inferior caval vein (**G**–**I**) and failure of the azygos vein and thoracic aorta timeline (**J**–**L**) was counteracted at 30 min (**G**,**J**), 5 h (**H**,**K**) and 24 h (**I**,**L**) ligation time period. Illustrative presentation included BPC 157 (10 ng/kg) or saline (5 mL/kg) as an intragastric administration immediately following ligation, as an early regimen. A similar presentation also included the effect of the delayed regimen.

**Figure 4:**
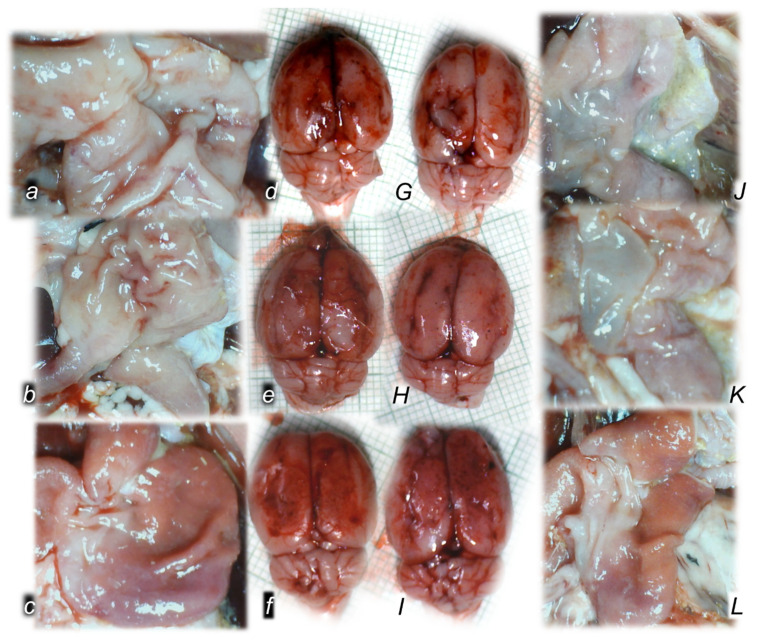
Stomach lesions and swollen brain in rats with occluded bile duct. Regular timeline (30 min (**a**,**d**), 5 h (**b**,**e**) and 24 h (**c**,**f**) ligation time period) of the stomach lesions’ presentation (**a**–**c**) and swollen brain (**d**–**f**) in controls. Upon BPC 157 therapy and recruitment of the defensive pathways, brain swelling (0–30 min (**G**), 0–5 h (**H**), 0–24 h (**I**) application sacrifice period) and gastric lesions (0–30 min (**J**), 0–5 h (**K**), 0–24 h (**L**) application sacrifice period) were counteracted. Illustrative presentation included BPC 157 (10 ng/kg) or saline (5 mL/kg) as an intragastric administration immediately following ligation, as an early regimen. A similar presentation also included the effect of the delayed regimen.

**Figure 5:**
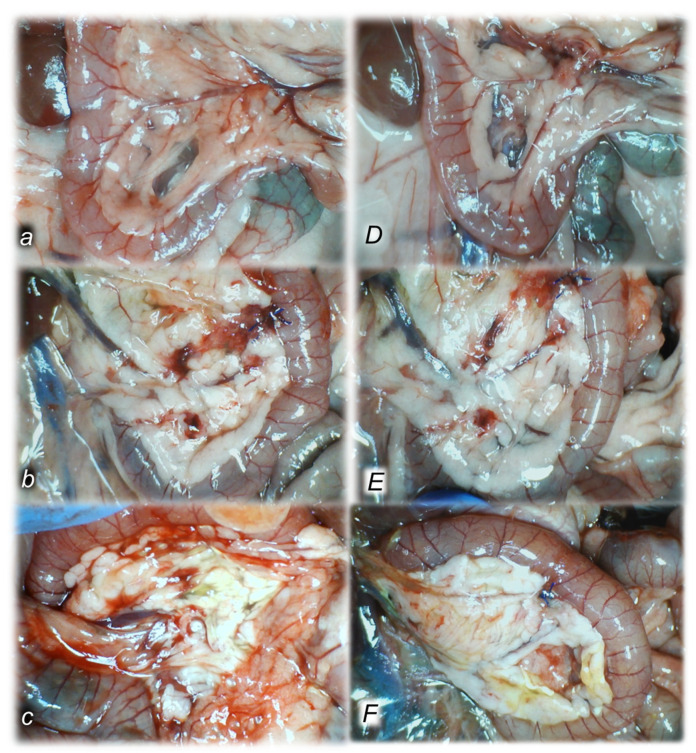
Regular timeline (30 min (**a**,**D**), 5 h (**b**,**E**) and 24 h (**c**,**F**) ligation time period) of disturbed pancreas presentation in controls (**a**–**c**). Upon BPC 157 therapy and recruitment of the defensive pathways, pancreas swelling and lesions (**D**–**F**) at 30 min (**D**), 5 h (**E**) and 24 h (**F**) ligation time period were counteracted. Illustrative recovered presentation included BPC 157 (10 ng/kg) or saline (5 mL/kg) as an intragastric administration, either immediately following ligation, as an early regimen (0–30 min (**D**), 0–24 h (**F**)), or as delayed regimen (4.30 h–5 h (**E**) application sacrifice period).

As a follow-up to the attenuation of blood pressure disturbances, peripherally and centrally, activation of the collateral pathway may compensate for major vessel failure and blood stasis observable with progressing thrombosis, which was fully counteracted in all veins and arteries investigated, peripherally and centrally. Consequently, the activated defensive response may be seen indicatively with the particular effects of BPC 157 on the relative volume of the congested vessels (superior mesenteric vein and inferior caval vein, from the trapped volume, congested liver and lung), dilated heart and collapsed vessels (azygos vein not functioning), and swollen brain (Figure 2). BPC 157 may decrease the increased relative volume of the superior mesenteric vein and inferior caval vein, increase the failed volume of the azygos vein, and bring these vessels and heart presentation close to normal vessel and heart presentation. Evidently, as a particular effect on blood vessels, congestion was reduced by the activation of the collateral bridging pathway, i.e., the azygos vein (Figure 2 and Figure 3), as BPC 157 increased the azygos vein’s relative volume (Figure 2 and Figure 3), combining the inferior caval vein and left superior caval vein to re-establish blood flow. Finally, as additional proof, the brain swelling and increased volume (associated with considerable brain injuries) (Figure 2 and Figure 4) was rapidly counteracted by BPC 157 administration, and induced a considerable decrease toward normal brain presentation. Interestingly, this effect was simultaneous with a comparable counteracting effect on the swollen pancreas (Figure 2 and Figure 5).

#### 3.1.4. Heart and ECG Disturbances

Commonly, the ligation of the bile duct was continuously timely along with the prolonged QTc intervals. However, the evidence shows that despite continuously maintained ligation of the bile duct, in all BPC 157-treated rats, QTc intervals’ prolongation was regularly absent. This occurred along with counteraction of the myocardial congestion (Figure 2).

#### 3.1.5. Serum Amylase Level

Without therapy, in all of the investigated ligation periods, the serum amylase levels were markedly increased. Likewise, BPC 157 therapy attenuated the increased serum amylase levels in all of the investigated ligation periods (Figure 2).

Additionally, at 0–24 h of the application sacrifice period (* *p* ˂ 0.05, at least, vs. control), BPC 157 decreased the increased AST (750 ± 55 (control), 330 ± 35 (μg) *, 350 ± 27 (ng) *), ALT (180 ± 36 (control), 70 ± 25 (μg) *, 80 ± 35 (ng) *) (U/L), total bilirubin (100 ± 15 (control), 40 ± 5 (μg) *, 35 ± 6 (ng) *), direct (60 ± 10 (control), 25 ± 5 (μg) *, 20 ± 5 (ng) *) and indirect (40 ± 5 (control), 15 ± 4 (μg) *, 15 ± 5 (ng) *) (μmol/L), creatinine (85 ± 8 (control), 50 ± 6 (μg) *, 55 ± 6 (ng) *) (μmol/L), urea (21 ± 3 (control), 6 ± 2 (μg) * 7 ± 3 (ng) *) (mmol/L) values, and counteracted thrombocytopenia (270 ± 25 (control), 670 ± 20 (μg) *, 770 ± 35 (ng) *).

### 3.2. A Perilous Syndrome Occurred Peripherally

#### 3.2.1. Pancreas, Gastrointestinal, Lung, Liver, Kidney, and Heart Lesions

The lesions are commonly noted to be vigorously expressed in the untreated rats with bile duct ligation, with an increased severity with ligation time; the lesion evidence in the affected organs may indicate particularity in the lesion course (Table 1). Thereby, the reduced severity of lesions in the pancreas, gastrointestinal tract and in the other organs by BPC 157 therapy may be seen as part of the cause-effect therapeutic course along with the reduced intracranial (superior sagittal sinus), portal and caval hypertension, and reduced aortal hypotension (Figure 1, Section 3.1.1). Together, this may suggest an immediate impact of the activated collateral pathway (i.e., 0–30 min and 4.30 h–5 h ligation period) followed by a sustained beneficial effect (0–24 h and 5 h–24 h ligation period) (Figure 2, Figure 3, Figure 4 and Figure 5).

##### Pancreas

Early regimen, period 0–30 min.

**Figure 6:**
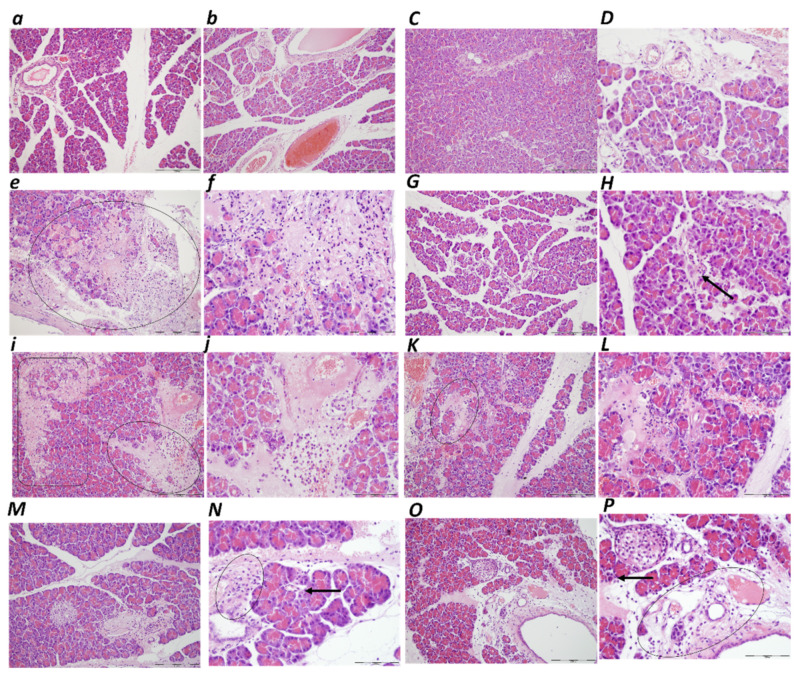
Pancreas lesion illustrative microscopic presentation in rats with obstructed bile duct (**a**–**P**) at the end of the ligation time periods of 30 min, 5 h, and 24 h; as an early regimen at 0–30 min (**a**–**D**), 0–5 h (**e**–**H**), and 0–24 h (**i**–**L**) application sacrifice period; and as a delayed regimen at 4.30 h–5 h (**M**,**N**) and 5 h–24 h (**O**,**P**) application sacrifice period in controls (**a**,**b**,**e**,**f**,**i**,**j**) and BPC 157-treated rats (**C**,**D**,**G**,**H**,**K**,**L**–**P**). (**a**,**b**) (Control, 0–30 min) Diffuse edema of interlobar septe, interlobular septe, interacinal spaces and focal expansion of intercellular spaces. Perivascular infiltration 6–10 leukocytes/HPF. (**C**,**D**) (BPC 157, 0–30 min) Focal edema of interlobar septe. No acinar necrosis, hemorrage, fat necrosis, inflammation or perivascular infiltrate. (**e**,**f**) (Control, 0–5 h) Diffuse edema of interlobar septe and interacinal spaces, and focal expansion of intercellular spaces, a diffuse occurrence of 5–10 necrotic acinar cells, a focal occurrence of 11–16 necrotic cells/HPF (black arrow), up to 2 foci of hemorrhage, up to 4 foci of fat necrosis (marked area), and perivascular infiltration 16–20 leukocytes/HPF. (**G**,**H**) (BPC 157, 0–5 h) Focal edema of interlobar septae. Focal occurence of 1–4 necrotic acinar cells/HPF (black arrow). No hemorrage and fat necrosis. Perivascular infiltration 2–5 leukocytes/HPF. (**i**,**j**) (Control, 0–24 h) Diffuse edema of interlobar septe, interlobular septe, interacinal spaces and focal expansion of intercellular spaces. More than 16 necrotic acinar cells/HPF (extensive confluent necrosis) (marked area). More than 8 foci of hemorrhage. Up to 7 foci of hemorrage and fat necrosis (marked area). Perivascular infiltration >30 leukocytes/HPF. (**K**,**L**) (BPC 157, 0–24 h) Focal edema of interlobar septe. Diffuse occurence of 1–4 necrotic acinar cells and focal occurrence of 15–10 necrotic cells/HPF. One focus of hemorrhage or fat necrosis (marked area). Perivascular infiltration 6–10 leukocytes/HPF. BPC 157 delayed regimens (**M**–**P**). (**M**,**N**) (BPC 157, 4.30 h–5 h) Focal edema of interlobar septe. Diffuse occurrence 5–10 necrotic cells/HPF (black arrow) and focal occurrence 11–16 necrotic cells/HPF. Up to one focus of hemorrhage, and up to 7 foci of fat necrosis, mostly in the pancreatic tail, while pancreatic head was not affected with fat necrosis. Perivascular infiltration >30 leukocytes/HPF (marked area). (**O**,**P**) (BPC 157, 5 h–24 h) Focal edema of interlobar septe. Diffuse occurrence 5–10 necrotic cells/HPF (black arrow) and focal 11–16 necrotic cells/HPF. Up to one focus of hemorrhage, and up to 7 foci of fat necrosis, mostly in the pancreatic tail, while pancreatic head was not affected with fat necrosis. Perivascular infiltration >30 leukocytes/HPF (marked area). (HE staining; magnification 200×; scale bar 200 μm; (**a**,**C**,**e**,**G**,**i**,**K**,**M**,**O**); (HE staining; magnification 400×; scale bar 100 μm; (**b**,**D**,**f**,**H**,**j**,**L**,**N**,**P**).

There were separate hemorrhagic zones and/or foci of necrosis, with the largest diameters in controls, while only edema was present in the pancreas of the BPC 157-treated rats. Microscopically, we noted in controls a diffuse edema of interlobar septe, interlobular septe, interacinal spaces, focal expansion of intercellular spaces, and perivascular infiltration of 6–10 leukocytes/HPF. Contrarily, BPC 157-treated rats with the early regimen presented only a focal edema of interlobar septe and no acinar necrosis, hemorrhage, fat necrosis, inflammation, or perivascular infiltrate (Figure 6).

Early regimen, period 0–5 h.

There were separate hemorrhagic zones and/or foci of necrosis, with the largest diameters in controls while in the BPC 157-treated rats there were mostly only edema in the pancreas. Microscopically, in controls, there was a diffuse edema of interlobar septe and interacinal spaces, focal expansion of intercellular spaces, a diffuse occurrence of 5–10 necrotic acinar cells, a focal occurrence of 11–16 necrotic cells/HPF, up to 2 foci of hemorrhage, up to 4 foci of fat necrosis, and perivascular infiltration of 16–20 leukocytes/HPF. Contrarily, early BPC 157 treatment had a marked beneficial effect, with only a focal edema of interlobar septa, a focal occurrence of 1–4 necrotic acinar cells/HPF, no hemorrhage and fat necrosis, and perivascular infiltration of 2–5 leukocytes/HPF (Figure 6).

Early regimen, period 0–24 h.

There were confluent and diffuse hemorrhagic zones and/or foci of necrosis, with the largest diameters in controls while BPC 157 exhibited separate hemorrhagic zones and/or foci of necrosis. Microscopically, controls presented increased lesion severity. They expressed a diffuse edema of interlobar septe, and interlobular septae, and focal expansion of intercellular spaces, more than 16 necrotic acinar cells/HPF (extensive confluent necrosis), more than eight foci of hemorrhage, up to seven foci of fat necrosis, and perivascular infiltration of >30 leukocytes/HPF. Contrarily, an early BPC 157 treatment had a marked beneficial effect until the end of the experiment, presenting markedly less lesions. Only a focal edema of interlobar septe, a diffuse occurrence of 1–4 necrotic acinar cells, a focal occurrence of 15–10 necrotic cells/HPF, up to one focus of hemorrhage and fat necrosis, and perivascular infiltration of 6–10 leukocytes/HPF was presented (Figure 6).

Delayed regimen, period 4.30 h–5 h.

There were separate hemorrhagic zones and/or foci of necrosis, with the largest diameters in controls. Contrarily, in the BPC 157-treated rats, there were mostly only edema in the pancreas. Microscopically, controls presented a diffuse edema of interlobar septe, interlobular septe, interacinal spaces, focal expansion of intercellular spaces, a diffuse occurrence of 5–10 necrotic acinar cells and a focal occurrence of 11–16 necrotic cells/HPF, up to four foci of hemorrhage, up to six foci of fat necrosis, and perivascular infiltration of 6–10 leukocytes/HPF in the 4.30 h–5 h assessment period (Figure 6).

Contrarily, in BPC 157-treated rats, we noted a focal edema of interlobar septe, a diffuse occurrence of 5–10 necrotic cells/HPF, a focal occurrence of 11–16 necrotic cells/HPF, up to one focus of hemorrhage, and up to seven foci of fat necrosis, mostly in the pancreatic tail, while the pancreatic head was not affected with fat necrosis. A perivascular infiltration of 16–20 leukocytes/HPF was found (Figure 6).

Delayed regimen, period 5 h–24 h.

There were confluent and diffuse hemorrhagic zones and/or foci of necrosis, with the largest diameters in controls. Contrarily, BPC 157 treatment exhibited separate hemorrhagic zones and/or foci of necrosis. Microscopically, a diffuse edema of interlobar septe, interlobular septe, interacinal spaces, diffuse expansion of intercellular spaces, more than 16 necrotic acinar cells/HPF (extensive confluent necrosis), more than eight foci of hemorrhage and fat necrosis, perivascular infiltration >35 leukocytes/HPF, and confluent microabscesses were found in the 5 h–24 h assessment period in the control group (Figure 6).

In BPC 157-treated rats, a focal edema of interlobar septe was noted along with diffuse occurrence of 5–10 necrotic cells/HPF, focal occurrence of 11–16 necrotic cells/HPF, up to one focus of hemorrhage, and up to seven foci of fat necrosis, mostly in the pancreatic tail, while the pancreatic head was not affected with fat necrosis. A perivascular infiltration >30 leukocytes/HPF was found in the 5 h–24 h assessment period (Figure 6).

##### Heart

**Figure 7:**
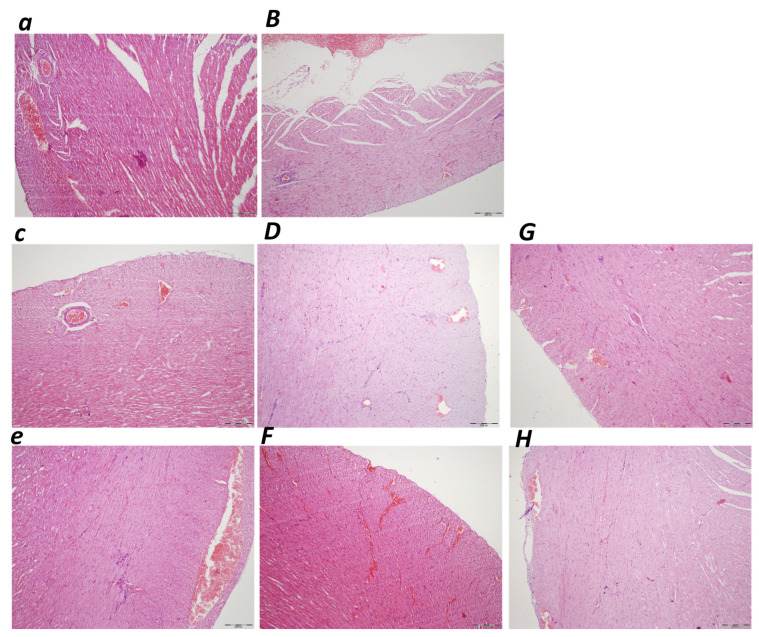
Heart congestion illustrative microscopic presentation (HE staining; magnification 100×; scale bar 200 μm) in rats with obstructed bile duct (**a**–**H**) at the end of the ligation time period of 30 min, 5 h, and 24 h; as an early regimen at 0–30 min (**a**,**B**), 0–5 h (**c**,**D**), and 0–24 h (**e**,**F**) application sacrifice period; and as delayed regimen at 4.30 h–5 h (**G**) and 5 h–24 h (**H**) application sacrifice period in controls (**a**,**c**,**e**) and BPC 157 treated rats (**B**–**E**). Marked myocardial congestion was consistently noted in controls at 30 min (**a**), 5 h (**c**) and 24 h (**e**) ligation time. For BPC 157 treatment, early regimen (**B**,**D**,**F**), no changes after 30 min ligation time (**B**), while mild myocardial congestion was found at 5 h (**D**) and 24 h (**F**) ligation time. In the delayed regimen (**G**,**H**), mild myocardial congestion was found at 4.30 h–5 h (**G**) and 5 h–24 h (**H**) application sacrifice period.

Heart congestion exhibited an illustrative microscopic presentation in rats with an obstructed bile duct at the end of the ligation time periods of 30 min, 5 h, and 24 h. Marked myocardial congestion was consistently noted in controls at 30 min, 5 h and 24 h ligation time (Table 1, Figure 7). With BPC 157, an early regimen resulted in no changes in the 30 min ligation time, and only mild myocardial congestion at 5 h (E) and 24 h ligation time. Likewise, a delayed BPC 157 regimen was combined with only mild myocardial congestion at 4.30 h–5 h and 5 h–24 h of the application sacrifice period.

##### Lung

**Figure 8:**
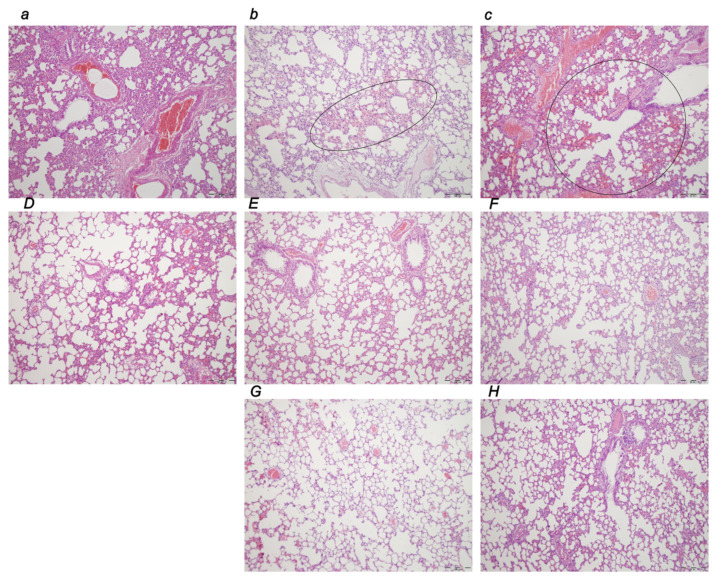
Lung tissue damage illustrative microscopic presentation (HE staining; magnification 100×; scale bar 200 μm) in rats with obstructed bile duct (**a**–**H**) at the end of the ligation time periods of 30 min, 5 h, and 24 h; as an early regimen at 0–30 min (**a**,**D**), 0–5 h (**b**,**E**), and 0–24 h (**c**,**F**) application sacrifice period; and as delayed regimen at 4.30 h–5 h (**G**) and 5 h–24 h (**H**) application sacrifice period in controls (**a**–**c**) and BPC 157-treated rats (**D**–**H**). In controls, marked lung parenchyma congestion was consistently noted from 30 min (**a**), in addition to intra-alveolar hemorrhage (marked area) at 5 h (**b**) and 24 h (**c**) ligation time. In the BPC 157 early regimen (**D**–**F**), there were no changes after 30 min ligation time (**D**), while mild lung tissue congestion was found at 5 h (**E**) and 24 h (**F**) ligation time. In the delayed regimen (**G**,**H**), mild lung tissue congestion was found at 4.30 h–5 h (**G**) and 5 h–24 h (**H**) application sacrifice period.

There was an illustrative microscopic presentation of the lung parenchyma congestion exhibited in rats with an obstructed bile duct at the end of the ligation time periods of 30 min, 5 h, and 24 h. Marked lung parenchyma congestion was consistently noted in controls at 30 min, 5 h and 24 h ligation time, along with intra-alveolar hemorrhage (Table 1, Figure 8). With BPC 157, an early regimen resulted in no changes after 30 min ligation time, and only mild lung congestion at 5 h (E) and 24 h of ligation time. Likewise, a delayed BPC 157 regimen was combined with only mild congestion at 4.30 h–5 h and 5 h–24 h of the application sacrifice period.

##### Liver

**Figure 9:**
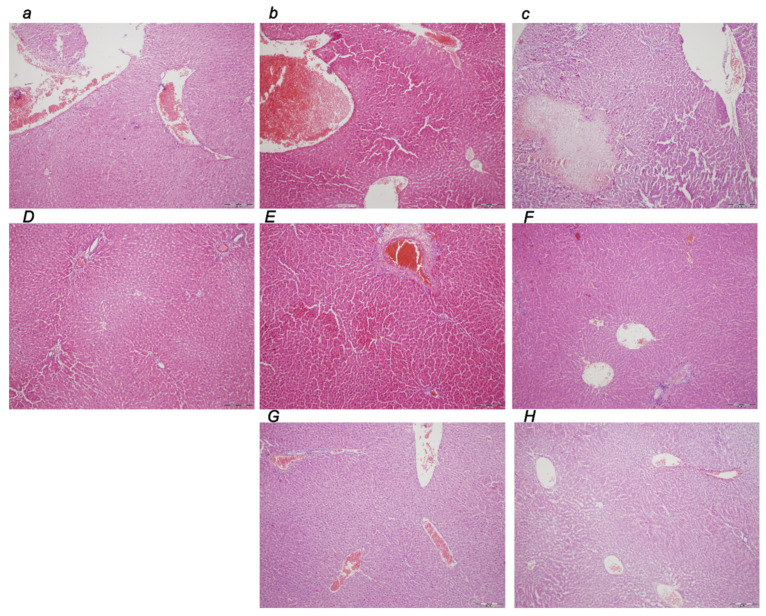
Liver tissue damage illustrative microscopic presentation (HE staining; magnification 100×; scale bar 200 μm) in rats with obstructed bile duct (**a**–**H**) at the end of the ligation time periods of 30 min, 5 h, and 24 h; as an early regimen at 0–30 min (**a**,**D**), 0–5 h (**b**,**E**), and 0–24 h (**c**,**F**) application sacrifice period; and as delayed regimen at 4.30 h–5 h (**G**) and 5 h–24 h (**H**) application sacrifice period in controls (**a**–**c**) and BPC 157-treated rats (**D**–**H**). In controls, there were marked dilatation and congestion of blood vessels in the portal tracts, central veins, and sinusoides at 30 min (**a**), 5 h (**b**), and 24 h (**c**) ligation time, along with the zones of confluent necrosis affecting liver lobuli and portal tract at 24 h (**c**) ligation time. In the BPC 157 early regimen (**D**–**F**), there were no changes after 30 min ligation time (**D**) while only mild dilatation and congestion of blood vessels in the portal tracts, central veins, and sinusoids was presented at 5 h (**E**) and 24 h (**F**) ligation time. In the delayed regimen (**G**,**H**), only mild dilatation and congestion of blood vessels in the portal tracts, central veins, and sinusoides were found at 4.30 h–5 h (**G**) and 5 h–24 h (**H**) application sacrifice period.

Liver tissue damage shows regular microscopic worsening in rats with an obstructed bile duct at the end of the ligation time periods of 30 min, 5 h, and 24 h (Table 1, Figure 9). At 30 min, 5 h, and 24 h of ligation time, there were marked dilatation and congestion of blood vessels in the portal tracts, central veins, and inusoids, along with the zones of confluent necrosis affecting liver lobuli and the portal tract, which occured at 24 h ligation time. Contrarily, the BPC 157 early regimen exhibited no changes after 30 min ligation time, while only mild dilatation and congestion of blood vessels in the portal tracts, central veins, and sinusoides was found at 5 h and 24 h (F) of ligation time. Likewise, with the delayed regimen, only mild dilatation and congestion of blood vessels in the portal tracts, central veins, and sinusoides were noted at 4.30 h–5 h and 5 h–24 h of the application sacrifice period.

##### Kidney

**Figure 10:**
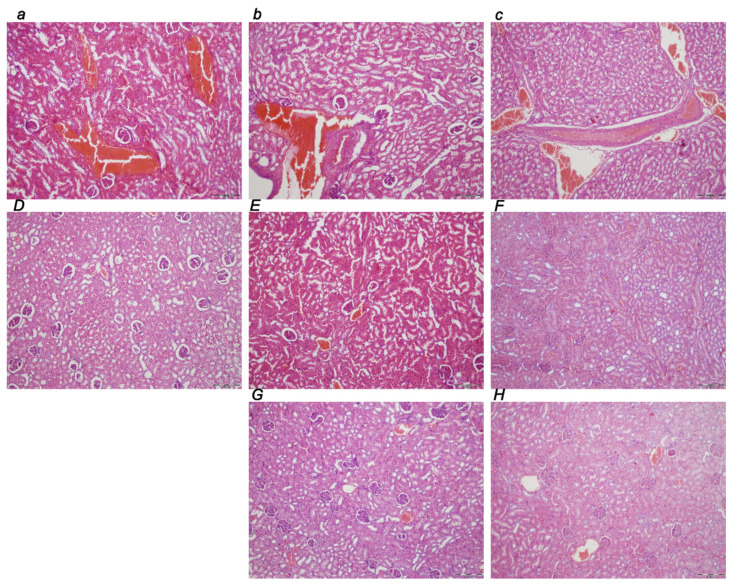
Kidney tissue damage illustrative microscopic presentation (HE staining; magnification 100×; scale bar 200 μm) in rats with obstructed bile duct (**a**–**H**) at the end of the ligation time periods of 30 min, 5 h, and 24 h; as an early regimen at 0–30 min (**a**,**D**), 0–5 h (**b**,**E**), and 0–24 h (**c**,**F**) application sacrifice period; and as delayed regimen at 4.30 h–5 h (**G**) and 5 h–24 h (**H**) application sacrifice period in controls (**a**–**c**) and BPC 157-treated rats (**D**–**H**). In controls, marked dilatation and congestion of blood vessels in the kidney tissue as well as glomeruli were found at 30 min (**a**), 5 h (**b**), and 24 h (**c**) ligation time. In the BPC 157 early regimen (**D**–**F**), there were no changes after 30 min ligation time (**D**) while only mild dilatation and congestion of blood vessels appeared at 5 h (**E**) and 24 h (**F**) ligation time. In the delayed regimen (**G**,**H**), only mild dilatation and congestion of blood vessels appeared at 4.30 h–5 h (**G**) and 5 h–24 h (**H**) application sacrifice period.

Marked dilatation and congestion of blood vessels in the kidney tissue as well as glomeruli were consistently noted at 30 min, 5 h, and 24 h (c) of ligation time (Table 1, Figure 10). Contrarily, the BPC 157 early regimen showed no changes at the 30 min ligation time period while only mild dilatation and congestion of blood vessels appeared at 5 h and 24 h of ligation time. Likewise, the delayed regimen resulted in only mild dilatation and congestion of blood vessels at 4.30 h–5 h and 5 h–24 h application sacrifice period.

##### Stomach

**Figure 11:**
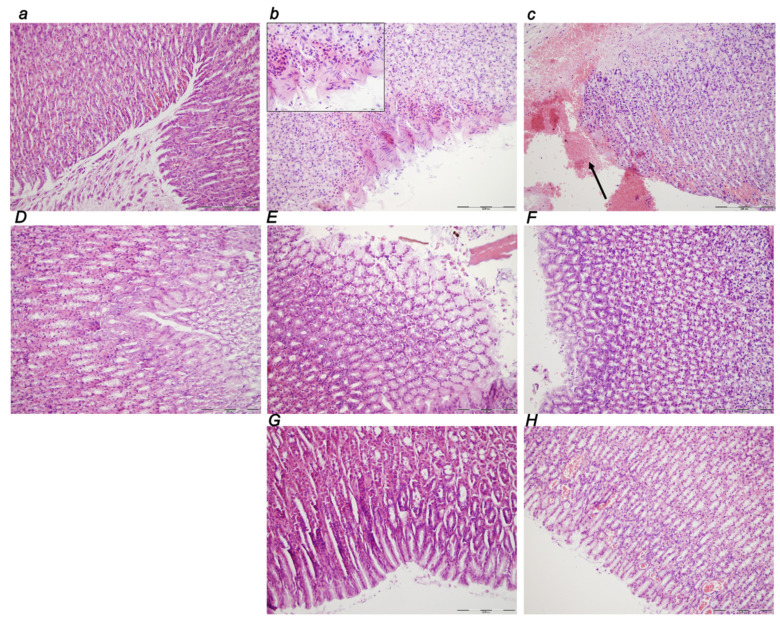
Stomach tissue damage illustrative microscopic presentation (HE staining; magnification 100×; scale bar 200 μm) in rats with obstructed bile duct (**a**–**H**) at the end of the ligation time periods of 30 min, 5 h, and 24 h; as an early regimen at 0–30 min (**a**,**D**), 0–5 h (**b**,**E**), and 0–24 h (**c**,**F**) application sacrifice period; and as delayed regimen at 4.30 h–5 h (**G**) and 5 h–24 h (**H**) application sacrifice period in controls (**a**–**c**) and BPC 157-treated rats (**D**–**H**). In controls, marked congestion of the stomach wall at 30 min (**a**), 5 h (**b**), and 24 h (**c**) ligation time was observed, along with hemorrhagic erosions (black arrow) and intramucosal neutrophilic inflammatory infiltration (rectangular marked area; magnification 400×; scale bar 50 μm) noted at 5 h and 24 h. In the BPC 157 early regimen (**D**–**F**), no changes appeared at 30 min (**D**), 5 h (**E**) and 24 h (**F**) ligation time. In the delayed regimen (**G**,**H**), no changes appeared at 4.30 h–5 h (**G**) while only mild congestion appeared at 5 h–24 h (**H**) application sacrifice period.

In controls, marked congestion of the stomach wall consistently occurred at 30 min, 5 h, and 24 h of ligation time (Table 1, Figure 11). Hemorrhagic erosions and intramucosal neutrophilic inflammatory infiltration were noted at 5 h and 24 h. The BPC 157 early regimen showed no changes at 30 min, 5 h and 24 h of ligation time. In the delayed regimen (Figure 11G,H), no changes appeared at 4.30 h–5 h while only mild congestion occurred at 5 h–24 h application sacrifice period.

##### Small Intestine

**Figure 12:**
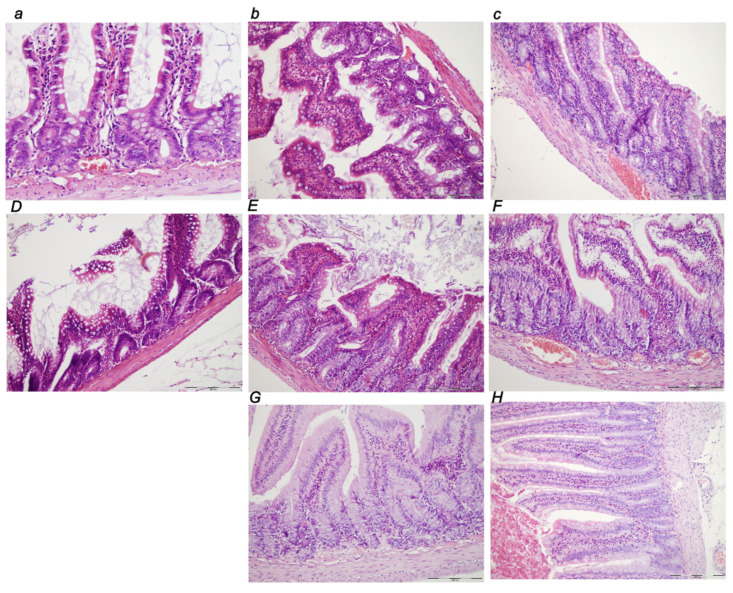
Small intestine damage illustrative microscopic presentation (HE staining; magnification 100×; scale bar 200 μm) in rats with obstructed bile duct (**a**–**H**) at the end of the ligation time periods of 30 min, 5 h, and 24 h; as an early regimen at 0–30 min (**a**,**D**), 0–5 h (**b**,**E**), and 0–24 h (**c**,**F**) application sacrifice period; and as delayed regimen at 4.30 h–5 h (**G**) and 5 h–24 h (**H**) application sacrifice period in controls (**a**–**c**) and BPC 157-treated rats (**D**–**H**). In controls, we consistently noted marked congestion at 30 min (**a**), 5 h (**b**), and 24 h (**c**) ligation time, more prominent at 24 h (**c**). In the BPC 157 early regimen (**D**–**F**), no changes appeared at 30 min (**D**), or 5 h (**E**), and mild congestion at 24 h (**F**) ligation time. In the delayed regimen (**G**,**H**), there was no change at 4.30 h–5 h (**G**) and only mild congestion appeared at 5 h–24 h (**H**) application sacrifice period.

In the small intestine, there was marked congestion at 30 min, 5 h, and 24 h of ligation time, being more prominent at 24 h. The BPC 157 early regimen showed no changes at 30 min, and 5 h, and only mild congestion at 24 h of ligation time. The delayed regimen showed no change at 4.30 h–5 h, and only mild congestion at 5 h–24 h of the application sacrifice period (Figure 12).

##### Colon

**Figure 13:**
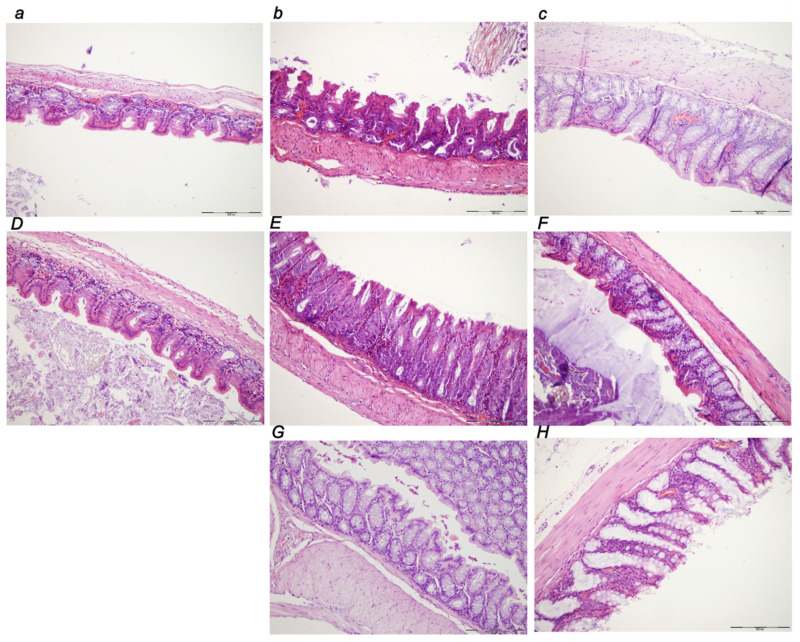
Large intestine damage illustrative microscopic presentation (HE staining; magnification 100×; scale bar 200 μm) in rats with obstructed bile duct (**a**–**H**) at the end of the ligation time periods of 30 min, 5 h, and 24 h; as an early regimen at 0–30 min (**a**,**D**), 0–5 h (**b**,**E**), and 0–24 h (**c**,**F**) application sacrifice period; and as delayed regimen at 4.30 h–5 h (**G**) and 5 h–24 h (**H**) application sacrifice period in controls (**a**–**c**) and BPC 157-treated rats (**D**–**H**). In controls, marked congestion appeared at 30 min (**a**), 5 h (**b**), and 24 h (**c**) ligation time, more prominent at 24 h (**c**). In the BPC 157 early regimen (**D**–**F**), no changes appeared at 30 min (**D**), 5 h (**E**) and mild congestion at 24 h (**F**) ligation time. In the delayed regimen (**G**,**H**), there was no change at 4.30 h–5 h (**G**) and only mild congestion appeared at 5 h–24 h (**H**) application sacrifice period.

In the large intestine, there was marked congestion at 30 min, 5 h, and 24 h ligation time, being most prominent at 24 h. The BPC 157 early regimen provided no changes at 30 min, and 5 h, and only mild congestion at 24 h of ligation time. The delayed regimen had no change at 4.30 h–5 h, and only mild congestion at 5 h–24 h of the application sacrifice period (Figure 13).

### 3.3. A Perilous Syndrome Occurred Centrally

#### 3.3.1. Brain Lesions, Cerebral and Cerebellar Cortex, Hypothalamus/Thalamus, and Hippocampus

Without therapy, the consistently downhill course of ligation of the bile duct at its entry into the duodenum in rats led to multiple organ lesions, widespread thrombosis, disturbed ECG and blood pressure, portal and caval hypertension, aortal hypotension, and in particular, intracranial (superior sagittal sinus) hypertension. From the immediate ligation period, the increased intracranial (superior sagittal sinus) hypertension coincided with a consistently swollen brain, severe brain edema and congestion, large intracerebral hemorrhage in the fronto-parietal area, and intraventricular hemorrhage in the central and lateral ventricles. Contrarily, reduced intracranial (superior sagittal sinus) hypertension (and eliminated portal and caval hypertension) with BPC 157 therapy counteracted the brain swelling with only mild brain edema and congestion, lesser brain hemorrhage, and no intraventricular hemorrhage.

#### 3.3.2. Brain Damage

**Figure 14:**
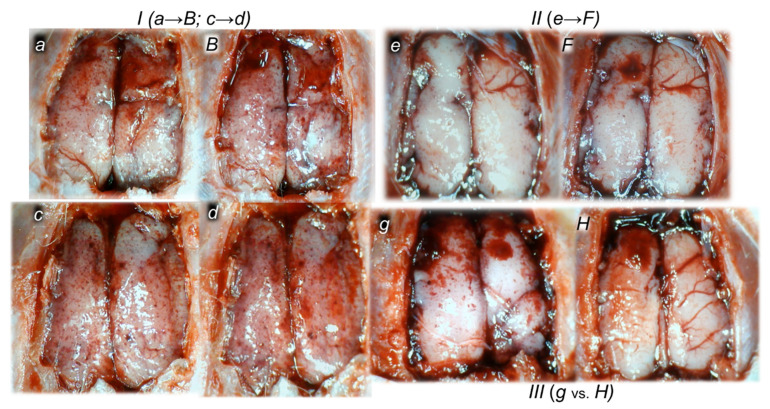
Gross presentation of the brain changes in rats with obstructed bile duct (**a**–**H**). Swollen brain (**a**,**c**–**e**,**g**) and brain swelling (saline application (**d**,**g**)), but counteracted by BPC 157 therapy (**B**,**F**,**H**) in rats with obstructed bile duct. I. Swollen brain before medication ((**a**,**c**) (immediate ligation-time)) and BPC 157 10 ng/kg intragastric administration, given immediately, ligation time 0, and effect assessed immediately thereafter; (**B**) the effect of the saline intragastric administration (**d**) assessed immediately (**d**) (worsened swelling due to uninterrupted progress of the injurious course). II. Swollen brain before medication (**e**) (4.30 h ligation time) and BPC 157 10 ng/kg intragastric administration, given at 4.30 h ligation time 0, and effect assessed immediately thereafter (**F**). III. Swollen brain (**g**) at 24 h ligation time, 24 h after saline (intragastric administration) given immediately after ligation. Swelling counteraction by BPC 157 therapy (**H**) at 24 h ligation time; BPC 157 10 ng/kg intragastric administration given at ligation time 0.

**Figure 15:**
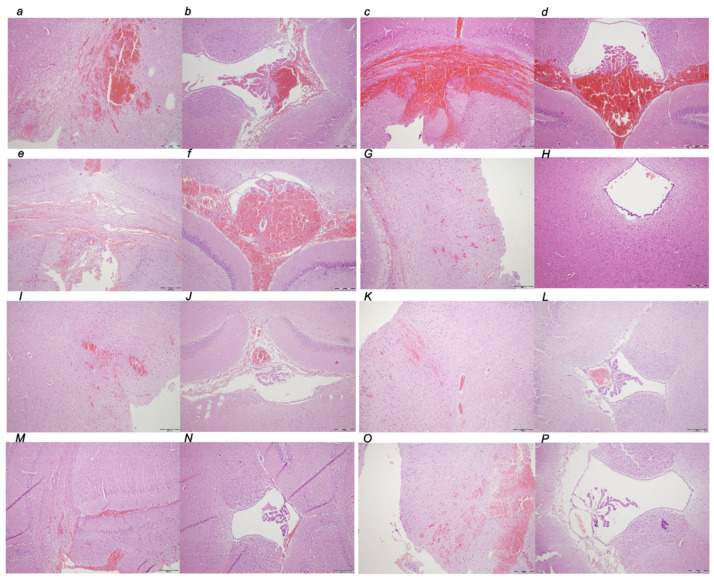
Brain hemorrhage lesion illustrative microscopic presentation in rats with obstructed bile duct (**a**–**P**) at the end of the ligation time periods of 30 min, 5 h, and 24 h; as an early regimen at 0–30 min (**a**,**b**,**G**,**H**), 0–5 h (**c**,**d**,**I**,**J**), and 0–24 h (**e**,**f**,**K**,**L**) application sacrifice period; and as delayed regimen at 4.30 h–5 h (**M**,**N**) and 5 h–24 h (**O**,**P**) application sacrifice period in controls (**a**–**f**) and BPC 157-treated rats (**G**–**P**). Control (**a**–**f**); (**a**,**b**) (0–30 min); (**c**,**d**) (0–5 h); (**e**,**f**) (0–24 h). BPC 157 regimens (**G**–**P**). Early regimens (**G**–**L**); (**G**,**H**) (0–30 min); (**I**,**J**) (0–5 h); (**K**,**L**) (0–24 h). Delayed regimens (**M**–**P**); (**M**,**N**) (4.30 h–5 h); (**O**,**P**) (5 h–24 h). (HE staining, magnification 100×; scale bar 200 μm). Control group. (**a**,**c**,**e**). Large areas of intracerebral hemorrhage in the corpus callosum, amygdala, and neocortex. (**b**,**d**,**f**). Intraventricular hemorrhage in the 3rd ventricle; BPC 157 group. (**G**,**I**,**K**,**M**,**O**). Minor intracerebral hemorrhage of the corpus callosum and neocortex. (**H**,**J**,**L**,**N**,**P**). No hemorrhage in the 3rd ventricle.

**Figure 16:**
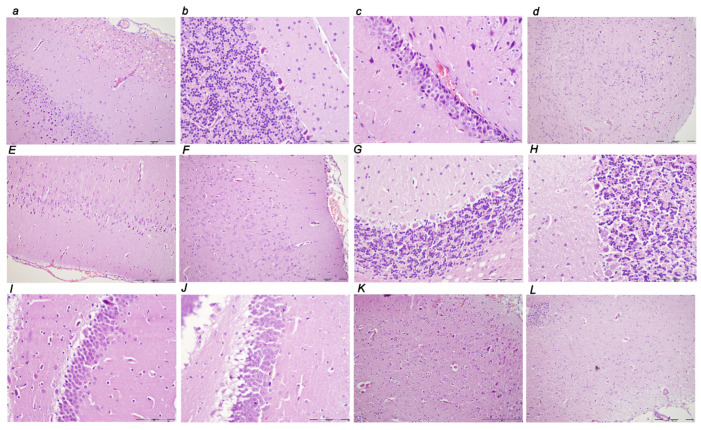
Neuropathological changes of cerebral area (**a**,**E**,**F**), cerebellar cortex (**b**,**G**,**H**), hyothalamus (**c**,**I**,**J**), and hippocampus (**d**,**K**,**L**) (HE staining; magnification 400×; scale bar 50 μm) in rats with obstructed bile duct (**a**–**L**) at the end of the ligation time period of 24 h. Controls. Necrosis of superficial neocortical areas of the cerebral tissue in control rats following karyopyknotic cells in the cerebral cortex (**a**), moderate karyopyknosis and degeneration of Purkinje cells of the cerebellar cortex (**b**), marked congestion and edema with marked karyopyknosis of pyramidal cells of the hippocampus (**c**), marked congestion and edema with marked karyopyknosis of pyramidal cells of the hypothalamus (**d**) at 24 h ligation time. BPC 157. No necrosis and only few karyopyknotic cells in cerebral cortex was found with early regimen 0–24 h (**E**) and delayed regimen at 5 h–24 h (**F**). No karyopyknotic cells in cerebellar cortex with early regimen 0–24 h (**G**). Only few karyopyknotic and degenerated Purkinje cells of the cerebellar cortex was found in the delayed regimen at 5 h–24 h (**H**). Only rare karyopyknotic cells in hippocampal area with early regimen 0–24 h (**I**) and delayed regimen at 5 h–24 h (**J**). Only rare karyopyknotic cells in hypothalamic area with early regimen 0–24 h (**K**) and delayed regimen at 5 h–24 h (**L**).

A pronounced edema and congestion were visible in the brain tissue in all four assessment time periods (i.e., 0–30 min, 0–5 h, 0–24 h, 4.30 h–5 h, 5 h–24 h) in the control group. A pronounced intracerebral hemorrhage involving larger areas of brain tissue was observed, affecting areas of the corpus callosum, amygdala, thalamus, neocortex, and striatum. Intraventricular hemorrhage involving the third and lateral ventricles was also seen (Table 2, Figure 14, Figure 15 and Figure 16).

In contrast, in the BPC 157 group, mild edema and congestion were visible in the brain tissue in all four assessment time periods (i.e., 0–30 min, 0–5 h, 0–24 h, 4.30 h–5 h, 5 h–24 h). Intracerebral hemorrhage affected only the corpus callosum, and superficial layers of the neocortex and amygdala. Few BPC 157 rats showed mild intraventricular hemorrhage in the third ventricle (0–5 h, 4.30 h–5 h, and 0 h–24 h).

After the assessment time periods of 0–24 h and 5 h–24 h, control rats presented severe neurodegenerative changes in the central nervous system. We noted an increased number of karyopyknotic cells markedly affecting all four regions, cerebral and cerebellar cortex, hypothalamus/thalamus and hippocampus. There was karyopyknosis and degeneration of the Purkinje cells of the cerebellar cortex, and a progression from mild to marked karyopyknosis of the cortical neurons and pyramidal cells of the hippocampus. These were all attenuated in BPC 157-treated rats.

### 3.4. Immunochemistry

**Figure 17:**
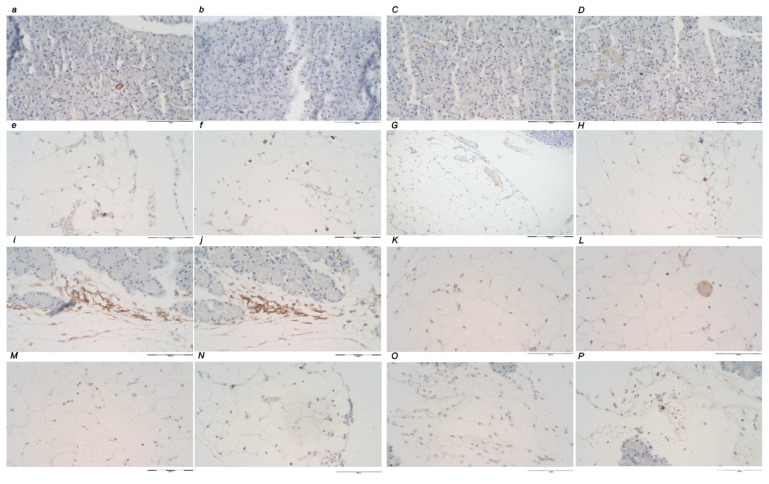
CD20- and CD3-positive cells’ illustrative immunochemistry presentation in rats with obstructed bile duct (**a**–**P**) at the end of the ligation time periods of 30 min, 5 h, and 24 h; as an early regimen at 0–30 min (**a**–**D**), 0–5 h (**e**–**H**), and 0–24 h (**i**–**L**) application sacrifice period; and as delayed regimen at 4.30 h–5 h (**M**,**N**) and 5 h–24 h (**O**,**P**) application sacrifice period in controls (**a**,**b**,**e**,**f**,**i**,**j**) and BPC 157-treated rats (**C**,**D**,**G**,**H**,**K**–**P**). “Hot spots” at 200× magnification. (**a**,**b**) (control, 0–30 min). Up to 1 cell CD20-positive and 2 cell CD3-positive lymphocytes were found within pancreatic lobuli. (**C**,**D**) (BPC 157, 0–30 min). Only up to 1 CD3-positive lymphocyte cell was found within pancreatic lobuli with no CD20 lymphocytes. (**e**,**f**) (control, 0–5 h). Up to 1 CD20-positive cell and 8 CD3-positive lymphocytes (**G**,**H**) (BPC 157, 0–5 h). Up to 1 CD20-positive cell, and no CD3-positive lymphocytes (**i**,**j**) (control, 0–24 h). Considerable number of CD20-positive cells and CD3-positive lymphocytes in the peripancreatic fat tissue. (**K**,**L**) (BPC 157, 0–24 h). Only up to 1 CD3-positive lymphocyte cell in the peripancreatic fat tissue, but no CD20 lymphocytes. BPC 157 delayed regimens (**M**–**P**). (**M**,**N**) (BPC 157, 4.30 h–5 h). Up to 1 CD20-positive cells, and no CD3-positive lymphocytes (**O**,**P**) (BPC 157, 5 h–24 h). Only up to 1 CD3-positive lymphocyte cell in the peripancreatic fat tissue, but no CD20 lymphocytes.

**Figure 18:**
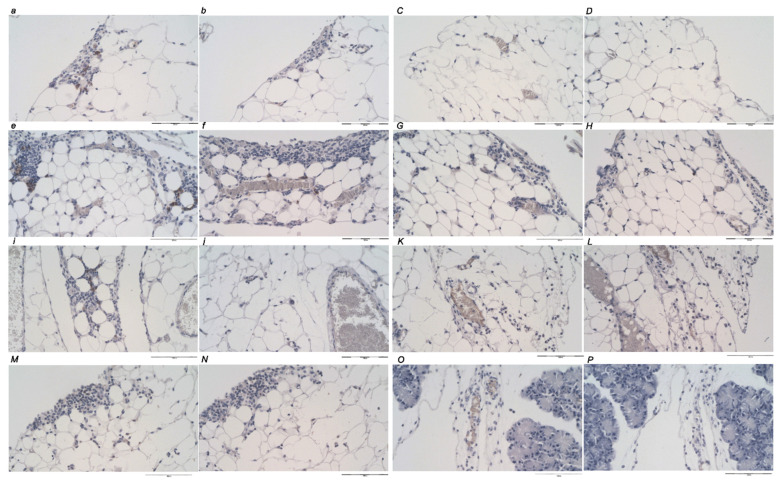
CD68- (PG-M1) and CD163-positive cells’ illustrative immunochemistry in rats with obstructed bile duct (**a**–**P**) at the end of the ligation time periods of 30 min, 5 h, and 24 h; as an early regimen at 0–30 min (**a**–**D**), 0–5 h (**e**–**H**), and 0–24 h (**i**–**L**) application sacrifice period; and as delayed regimen at 4.30 h–5 h (**M**,**N**) and 5 h–24 h (**O**,**P**) application sacrifice period in controls (**a**,**b**,**e**,**f**,**i**,**j**) and BPC 157-treated rats (**C**,**D**,**G**,**H**,**K**–**P**). “Hot spots” at 200× magnification. (**a**,**b**) (control, 0–30 min). Considerable number of positive CD68 (PG-M1)-positive macrophages, but no CD163-positive macrophages in the peripancreatic fat tissue. (**C**,**D**) (BPC 157, 0–30 min). No CD68- (PG-M1) and CD163-positive macrophages. (**e**,**f**) (control, 0–5 h). Considerable number of positive CD68- (PG-M1) and CD163-positive macrophages in the peripancreatic fat tissue. (**G**,**H**) (BPC 157, 0–5 h). Only up to 1 CD163-positive macrophage in the peripancreatic fat tissue, but no CD68 (PG-M1)-positive macrophages. (**i**,**j**) (control, 0–24 h). Considerable number of CD68 (PG-M1)-positive and only 1 CD163-positive macrophages in the peripancreatic fat tissue. (**K**,**L**) (BPC 157, 0–24 h). No CD68- (PG-M1) and CD163-positive macrophages. BPC 157 delayed regimens (**M**–**P**). (**M**,**N**) (BPC 157, 4.30 h–5 h). Only up to 1 CD163-positive macrophage in the peripancreatic fat tissue, but no CD68 (PG-M1)-positive macrophages. (**O**,**P**) (BPC 157, 5 h–24 h). Only up to 1 CD163-positive macrophage in the peripancreatic fat tissue, but no CD68 (PG-M1)-positive macrophages.

There is a distinctive difference between the control and treated animals regarding inflammatory cells (Figure 17 and Figure 18, Table 3). An increase in inflammatory infiltration, particularly CD3-positive lymphocytes and M1 CD68 (PG-M1)-positive macrophages, were observed in the control group, which follows the timeline of the inflammatory process. There was an increased number of M2 CD163-positive macrophages observed, lower than the number of M1 macrophages. This reflects the regular homeostasis of the inflammatory response to the tissue injury. Contrarily, in treated animals, there was none, or a distinctively mild nonspecific inflammation found, with only rare CD3-positive lymphocytes. There was no macrophage activation and barely one M2 CD163-positive macrophage in the “hot spot” area at 5 h.

In summary, after BPC 157 therapy, rats with a ligated bile duct exhibited no portal and caval hypertension, ameliorated aortal hypotension, markedly attenuated superior sagittal sinus hypertension, and no prolonged QTc interval. Additionally, venous and arterial thrombosis was attenuated, both peripherally and centrally, with reduced brain, heart, lung, liver, kidney, and gastrointestinal lesions, and counteracted acute pancreatitis, as either an initial or final result (severe intracerebral and intraventricular hemorrhage at the time when the pancreas exhibited only edema). The laboratory parameters improved and a particular inflammatory response was indicated. The key finding of an activated particular collateral pathway, i.e., the azygos vein, which combined the inferior caval vein and left superior vein to reorganize blood flow, might be responsible for the noted beneficial effects.

## 4. Discussion

Interestingly, for the acute pancreatitis and general disturbances (for review, see, i.e., [8,9,10]), it may be that the pancreas itself is relatively spared since pancreatic microcirculation in mild edematous pancreatitis is significantly increased, while only in the evolution of necrotizing pancreatitis may occur strongly reduced pancreatic capillary flow in conjunction with areas of capillary stasis [55]. Thereby, from an advanced therapy viewpoint, defining acute pancreatitis as particular (even antecedent) “occlusion-like” syndrome reveals only the innate vascular failure as a special target. This vascular failure might be similar to that created with the endothelium-damaging agents’ major intoxication [24,25], myocardial infarction [23], and maintained severe intra-abdominal hypertension [22], or those created with major vessel(s) occlusion [14,15,16,20], veins [15,16,20] or arteries [14], peripheral [14,15,16] and central [20]; however, all were consistently resolved by BPC 157 therapy [14,15,16,20,23,24,25]. Possibly also in acute pancreatitis and general disturbances (for review, see, i.e., [8,9,10]), the BPC 157 bypassing key, as a particular cytoprotection mediator, native and stable in human gastric juice, might bring the upgraded original endothelium/epithelium maintenance background to an observable principle (for review, see i.e., [1,2,3,4,5,6,7]). Evidently, the principle rapidly functions as promptly reestablished and reorganized blood flow (i.e., activated collateral circulation) when the upgraded minor vessel can take over the function of the injured major vessel and overwhelm the Virchow triad circumstances, which is otherwise commonly presented (for review, see i.e., [1,2,3,4,5,6,7]). Illustratively, instead of a completely failed azygos vein (i.e., that commonly noted in “occlusion-like” syndrome studies and Budd-Chiari syndrome [13,22,23,24,25]), there is a prompt and sustained activation of the azygos vein in the BPC 157-treated bile duct-ligated rats. Thus, as before in the “occlusion-like” syndrome studies or in the Budd-Chiari (suprahepatic inferior caval vein occlusion) occlusion-syndrome [13,22,23,24,25], there was direct blood delivery from the inferior caval vein to the superior caval vein to break the vicious cycle instantly. In support, the therapy’s beneficial effect in the rats with occluded bile ducts was relatively marked, as before in the “occlusion-like” syndrome and Budd-Chiari–syndrome studies [13,23,24,25]. In the bile duct-ligated rats as well as in the “occlusion-like” syndrome and Budd-Chiari–syndrome rats [13,22,23,24,25], there was a consistent counteraction of the intracranial (superior sagittal sinus) hypertension, portal and caval hypertension, aortal hypotension, and congested veins (i.e., reflecting trapped volume—congested inferior caval vein and superior mesenteric vein). In all of them [13,22,23,24,25], there was a reversal of the multiorgan failure (i.e., brain, heart, lung, liver, kidney, pancreas and gastrointestinal tract lesions, and progressing thrombosis, peripherally and centrally). Thus, there is consistent evidence of an upgraded venous system that might apply to the rats with ligated bile ducts, in particular. Consequently [13,22,23,24,25], there is a wide reversal of the shared effect of all the negative events. These might act peripherally (i.e., bile duct ligation), centrally (i.e., huge intraventricular hemorrhage), and then peripherally and centrally, and centrally and peripherally, along with the three interconnected body cavities, as specifically shown in the maintained intra-abdominal hypertension studies [22], as the disturbances may be rapidly transmitted from the periphery to centrally, and from centrally to the periphery, through the venous system. BPC 157 therapy might also effectively operate under the conditions of severe intra-abdominal hypertension, even grade III and grade IV [22].

Finally, there was a similar therapy outcome [13,22,23,24,25] observable in the bile duct-ligated rats given BPC 157 regimens (both doses, both intragastric bolus and continuous oral application in drinking water, both early regimen and delayed regimen); the principle might be equally successfully initiated during the ongoing injury course and affect the injury outcome at any stage. Together, there is common evidence for resolving vascular injuries in rats (including bile duct obstruction/acute pancreatitis) [11,12,13,14,15,16,17,18,19,20,21,22,23,24,25,26,27,28], and thereby, for the BPC 157 therapy’s effect on both the acute pancreatitis itself (i.e., improved gross and microscopy presentation, decreased amylase level) and the general concomitant “occlusion-like” syndrome.

However, there might be an indication as to how the particular counteraction with BPC 157 therapy might advance during a noxious course. Namely, the evidence may be indicative that at an early time point, the pancreas exhibited mostly edema only; the major pancreatitis lesions (i.e., acinar, fat necrosis, hemorrhage) regularly appeared in the later period [55]. This might favor the prime central early course, the brain swelling, lesions and huge hemorrhage as the most prominent damage; increased (superior sagittal sinus) hypertension might have thereby a decisive role. Additionally, BPC 157’s beneficial effects appear to be particularly appealing centrally. Illustratively, BPC 157 is very active in the rats with occluded superior sagittal sinus [20], as well as when given in the reperfusion in the rats with stroke, where BPC 157 therapy counteracted hippocampal ischemia/reperfusion injuries and functional and cognitive deficits [21]; when given in the rats with spinal cord compression, BPC 157 rapidly decreased spinal hematoma and increased function recovery [56,57]. In all of the vascular studies [14,15,16,20,22,23,24,25], as also in the rats with ligated bile ducts, there was a prominent reversal of the brain swelling shortly after BPC 157 therapy application along with a decrease in intracranial (superior sagittal sinus) hypertension. It is possible that the BPC 157 therapy’s effect might accommodate the particular central/peripheral and peripheral/central equation that may also take place in the bile duct-ligated rats. Namely, in analogy, we evidenced the occluded superior sagittal sinus-produced central disturbances (i.e., intracranial (superior sagittal sinus) hypertension) rapidly transmitted to the periphery (i.e., portal, caval hypertension, aortal hypotension, multiorgan lesions) [20]. Vice versa, direct occlusion of the superior mesenteric vessels, vein, artery, or vein and artery (i.e., portal, caval hypertension, aortal hypotension, peripheral lesions) rapidly induced the intracranial (superior sagittal sinus) hypertension, brain swelling, and lesions [14,15,16]. As this beneficial BPC 157 effect occurred in either circumstance, there was adequate resolution of the anatomical imbalance in venous drainage, which would thereby not occur [20]; centrally, a harmful inability to drain venous blood adequately for a given cerebral blood inflow without raising venous pressures was resolved, evidencing rapid counteraction of such venous and intracranial hypertension [20,58,59,60,61,62]. Notably, in the timeline’s causes and effects in the bile duct-ligated rats, the obtained frame (i.e., 0–30 min, 0–5 h, 4.30 h–5 h, 5 h–24 h, 0–24 h of the application sacrifice periods to verify consistency of the given therapy) might imply the timely advanced adverse effects of that shared syndrome, all and/or individual, into the essential presentation of the acute pancreatitis, and vice versa. This may indicate that the rapid initial presentation and subsequent injurious course is well presented. This may reveal the particular relationships (and therapy significance considering lesion counteraction in particular) and multidirectional network between the acute pancreatitis presentation and all other pathology targets. In particular relations, acute pancreatitis causing other organ lesions, we may characterize the brain swelling and lesions (i.e., intraventricular hemorrhage), heart dysfunction (i.e., prolonged QTc interval, congestion), lung lesions (i.e., hemorrhage), liver and kidney failure (i.e., severe congestion), and gastrointestinal lesions. Furthermore, there were widespread arterial and venous thrombosis, vein congestion (inferior caval vein and superior mesenteric vein) or failure (i.e., azygos vein), and severe intracranial (superior sagittal sinus), portal and caval hypertension and aortal hypotension. Thus, in this mutually interacting large damaging circuit in acute pancreatitis syndrome, the evidence of effective therapy that did counteract pancreatitis may indicate that it rapidly travels to affects all of the mentioned points, as we demonstrated with the BPC 157 therapy regimens. Illustratively, as a common beneficial effect of BPC 157 therapy, the pancreatic head was not affected by fat necrosis, there were no changes or only mild myocardial, lung, liver, kidney, stomach and intestinal congestion, and limited brain lesions (i.e., unlike extensive intraventricular hemorrhage in controls, only mild intraventricular hemorrhage in the third ventricle) in the BPC 157 rats. Such a pleiotropic effect (i.e., in the bile duct ligation/liver cirrhosis studies, with BPC 157 therapy, portal hypertension was either not developed or rapidly annihilated, depending on the given BPC 157′s regimen, and BPC 157 therapy might have counteracted liver fibrosis and portal hypertension [31]) was constant. As in other studies, there was counteraction of the brain [21], heart [63,64,65,66,67,68], lung [69,70,71,72], and liver lesions [31,73,74], liver, gastrointestinal and brain lesions [75,76,77,78,79,80], and kidney [14,15,16,20,22,23,24,25] and pancreas [29,30] lesions. In the original cytoprotection studies, pancreatitis was known as a particular target for the standard cytoprotective agent activity [81], combined with the stomach and duodenal lesions [29,30]. Thereby, the activated azygos vein [13,14,15,16,20,22,23,24,25] (rat azygos vein partially resembles the atrial myocardium [82]) may indeed be the BPC 157 bypassing key. As a BPC 157 therapy effect, the combined inferior caval vein and superior caval vein via direct blood delivery initiated the defensive response that made rats capable of enduring the bile duct ligation at its entry into the duodenum with a more preserved pancreas. Thus, the consequent noxious “occlusion-like” syndrome, occlusion syndrome and multiple organ failure was peripherally and centrally avoided [13,14,15,16,20,22,23,24,25], despite the permanent occlusion of the bile duct. Finally, with BPC 157 therapy Virchow was reversed, there was a reversal of arterial and venous thrombosis, a common effect that was rapid and sustained [11,12,13,14,15,16,20,22,23,24,25]. This means that in rats with bile duct ligation, the therapy reversed the stasis peripherally (note also the counteracted arrhythmias) and centrally, and re-established blood flow and largely rescued rats with ligated bile duct at its entry into the duodenum, thereby counteracting the intracranial (superior sagittal sinus) hypertension, portal and caval hypertension and aortal hypotension [11,12,13,14,15,16,20,22,23,24,25]. Thus, general multiorgan failure, in particular that of the acute pancreatitis, appears to be due to the constant lack of the activated collateral pathway and may be a special target for the therapy application.

Finally, although not specifically investigated in this study, we should mention that BPC 157 therapy, in addition to decreasing increased serum amylase, counteracted the increased ALT, AST, bilirubin, urea, and creatinine values. BPC 157 therapy counteracted marked thrombocytopenia (and thereby over-increased thrombocytes’ consumption, with increased (i.e., intraventricular) bleeding not occurring [11]). Likewise, in the BPC 157-treated rats, there was no inflammation, or a distinctively mild, nonspecific inflammation, with only rare CD3-positive lymphocytes. There was no macrophage activation, and barely one M2 CD163-positive macrophage was found in the “hot spot” area at 5 h; the significance of that modified response resulting from the therapy needs to be further defined. We would also further emphasize some additional supportive arguments, which were also not specifically investigated in this study. BPC 157 therapy intercepting vascular failure might be understood through a particular modulatory effect, the complex reorganizing of the blood flow by activating particular collateral (i.e., azygos vein) pathways that might compensate the failed circumstances created by the persisting noxious event (i.e., occluded bile duct). This might be a particular effect (i.e., activation of an alternative operating pathway, as when the left ovarian vein as the rescuing key for infrarenal occlusion-induced inferior vena cava syndrome in rats was ascribed to *Egr*, *Nos*, *Srf*, *Vegfr*, *Akt1*, *Plcɣ*, and *Kras* genes’ particular expression in the vessel [11]). In interacting with many molecular pathways, this might be particular BPC 157/NO system evidence (i.e., combining maintained endothelium function and maintained thrombocyte function—and thereby counteracting thrombocytopenia in bile duct-ligated rats as before in deep vein thrombosis studies [11], and counteracting thrombosis in all vascular studies [12,13,14,15,16,20,22,23,24,25])—with coagulation pathways not affected). Further arguments might include modulatory effects on the NO system (NO release’s—BPC 157 induced the NO release on its own—particular effect on NOS inhibition (i.e., opposed hypertension and pro-thrombotic effect of L-NAME) and particular effect on NO over-stimulation (i.e., opposed hypotension and anti-thrombotic, pro-bleeding effect of L-arginine) [39,40,41,42,43,44] controlling vasomotor tone and the activation of Src-Caveolin-1-eNOS pathway [48,49]). There may also be modulatory effects on the prostaglandins system [4,35] (BPC 157 counteracted NSAID toxicity (for review, see, i.e., [35]) and indomethacin-induced leaky gut syndrome in particular (for review, see, i.e., [4]). Consequently, the final clue may be that BPC 157 acts via increasing tight junction protein ZO-1 expression, and transepithelial resistance, inhibiting the mRNA of inflammatory mediators (iNOS, IL-6, IFNγ, and TNF-α), and increasing the expression of HSP 70 and 90, and antioxidant proteins, such as HO-1,NQO-1, glutathione reductase, glutathione peroxidase 2, and GST-pi, and counteracting leaky gut syndrome [4]. Considering this essential mechanism responsible for various severe systemic diseases, this might fully substantiate the significance of BPC 157 therapy in the realization of the recovery of the essential vascular failure in the rats with ligated bile ducts, as before in the other occlusion/“occlusion-like” syndromes [12,13,14,15,16,20,22,23,24,25,31], and thereby the full therapy effect. This free radical scavenger effect [4,11,12,13,14,15,16,23,24,31,36,37,38,83] occurs in both ischemic and reperfusion conditions in the various tissues (i.e., brain, colon, duodenum, cecum, liver, muscle, and veins) and plasma [4,11,12,13,14,15,16,23,24,31,36,37,38,83].

Finally, this special issue (i.e., bile duct occlusion, acute pancreatitis, presented with vascular failure, antecedent and concurrent occlusion-like syndrome, with multidirectional relation between the acute pancreatitis and other organs’ lesions) remains to be further elaborated. Likewise, the pentadecapeptide BPC 157 should be further investigated. The therapy involved an intragastric bolus, and continuous oral application in drinking water, activated azygos vein direct blood delivery, counteracted/attenuated the intracranial (superior sagittal sinus), portal and caval hypertension, aortal hypotension, multiorgan failure (brain, heart, lung, liver, kidney, stomach, and intestinal lesions), and progressing thrombosis. Thereby, we observed acute pancreatitis, improved gross and microscopy presentation, and decreased amylase levels that might be applied also to other acute pancreatitis-type induction. We used the bile duct occlusion, acute pancreatitis, occlusion-like syndrome, activated azygos vein collateral pathway, pentadecapeptide BPC 157, intragastric bolus, and continuous oral application in drinking water as a simple but useful key, and shared the same dose relation in all BPC 157/vascular studies (for review, see, i.e., [1,2,3,4,5,6]). This might be seen as a network of evidence for the physiological significance of the revealed BPC 157/vascular system interplay (i.e., BPC 157 was found in situ hybridization and immunostaining studies in humans to be largely distributed in tissues [84] and may have additional physiological regulatory roles [3,84]). Moreover, there is also a very safe BPC 157 profile (i.e., no adverse effects in clinical trials (ulcerative colitis, phase II); in toxicological studies, a lethal dose (LD1) could be not achieved) (for review see, i.e., [1,2,3,4,5,6,84]), a point recently confirmed in a large study conducted by Xu and collaborators [85]. Together, these findings (for review, see, i.e., [1,2,3,4,5,6,84]) might suggest BPC 157 therapy application for further vascular injuries, and appropriate use in acute pancreatitis therapy as well.

In addition, case reports and retrospective cohort studies have reported an association between acute pancreatitis and COVID-19 [86], while recently, due to the described effects, the stable gastric pentadecapeptide BPC 157 was suggested as a potential treatment for COVID-19 [7].

## Figures and Tables

**Table 1:** Pancreatitis, heart, lung, liver, kidney, stomach, small and large intestine lesion assessment in the bile duct-ligated rats following ligation (time 0) at sequential periods (application sacrifice) to illustrate the effect of both early treatment and delayed treatment following ligation. * *p* ˂ 0.05, at least, vs. control.

Regimens	Early Regimen			Delayed Regimen	
Periods	0–30 min	0–5 h	0–24 h	4.30 h–5 h	5 h–24 h
**Treatment**	**Pancreatitis (gross presentation scored 0–5, Min/Med/Max)**				
Control	1/2/2	2/2/3	4/4/5	2/2/3	4/4/5
BPC 157 10 μg/kg	0/1/1 *	1/1/2 *	2/2/3 *	1/1/2 *	2/2/3 *
BPC 157 10 ng/kg	0/1/1 *	1/1/2 *	2/2/3 *	1/1/2 *	2/2/3 *
	**Pancreatitis (microscopy scored 0–20, Min/Med/Max)**				
Control	4/4/5	12/13/13	19/20/20	12/13/13	19/20/20
BPC 157 10 μg/kg	0/0/1 *	1/1/2 *	4/4/4 *	7/7/8 *	11/12/12 *
BPC 157 10 ng/kg	0/0/1 *	1/1/2 *	4/4/4 *	7/7/8 *	11/12/12 *
	**Heart (scored 0–3, Min/Med/Max)**				
Control	2/2/2	2/2/2	3/3/3	2/2/2	3/3/3
BPC 157 10 μg/kg	0/0/0 *	1/1/1 *	1/1/1 *	1/1/1 *	1/1/1 *
BPC 157 10 ng/kg	0/0/0 *	1/1/1 *	1/1/1 *	1/1/1 *	1/1/1 *
	**Lung (scored 0–3, Min/Med/Max)**				
Control	2/2/2	3/3/3	3/3/3	3/3/3	3/3/3
BPC 157 10 μg/kg	0/0/0 *	0/0/0 *	1/1/1 *	1/1/1 *	1/1/1 *
BPC 157 10 ng/kg	0/0/0 *	0/0/0 *	1/1/1 *	1/1/1 *	1/1/1 *
	**Liver (scored 0–3, Min/Med/Max)**				
Control	2/2/2	2/2/2	3/3/3	2/2/2	3/3/3
BPC 157 10 μg/kg	0/0/0 *	1/1/1 *	1/1/1 *	1/1/1 *	1/1/1 *
BPC 157 10 ng/kg	0/0/0 *	1/1/1 *	1/1/1 *	1/1/1 *	1/1/1 *
	**Kidney (scored 0–3, Min/Med/Max)**				
Control	2/2/2	2/2/2	2/2/2	2/2/2	2/2/2
BPC 157 10 μg/kg	0/0/0 *	0/0/0 *	1/1/1 *	0/0/0 *	1/1/1 *
BPC 157 10 ng/kg	0/0/0 *	0/0/0 *	1/1/1 *	0/0/0 *	1/1/1 *
	**Stomach (sum of longest diameters, mm, means ± SD)**				
Control	5 ± 1	5 ± 1	10 ± 1	5 ± 1	10 ± 1
BPC 157 10 μg/kg	0 ± 0 *	0 ± 0 *	0 ± 0 *	0 ± 0 *	0 ± 0 *
BPC 157 10 ng/kg	0 ± 0 *	0 ± 0 *	0 ± 0 *	0 ± 0 *	0 ± 0 *
	**Stomach (scored 0–15, Min/Med/Max)**				
Control	5/5/5	8/8/8	5/5/5	8/8/8	5/5/5
BPC 157 10 μg/kg	0/0/0 *	0/0/0 *	2/2/2 *	0/0/0 *	0/0/0 *
BPC 157 10 ng/kg	0/0/0 *	0/0/0 *	2/2/2 *	0/0/0 *	0/0/0 *
	**Small intestine (scored 0–15, Min/Med/Max)**				
Control	4/4/4	4/4/4	5/5/5	4/4/4	5/5/5
BPC 157 10 μg/kg	0/0/0 *	0/0/0 *	2/2/2 *	0/0/0 *	2/2/2 *
BPC 157 10 ng/kg	0/0/0 *	0/0/0 *	2/2/2 *	0/0/0 *	2/2/2 *
	**Large intestine (scored 0–15, Min/Med/Max)**				
Control	2/2/2	2/2/2	4/4/4	2/2/2	4/4/4
BPC 157 10 μg/kg	0/0/0 *	0/0/0 *	2/2/2 *	0/0/0 *	1/1/1 *
BPC 157 10 ng/kg	0/0/0 *	0/0/0 *	2/2/2 *	0/0/0 *	1/1/1 *

**Table 2:** Brain lesion assessment in the bile duct-ligated rats following ligation (time 0) at sequential periods (application sacrifice) to illustrate the effect of both early treatment and delayed treatment following ligation. * *p* ˂ 0.05, at least, vs. control.

Regimens	Early Regimen			Delayed Regimen	
Periods	0–30 min	0–5 h	0–24 h	4.30 h–5 h	5 h–24 h
**Treatment**	**Cerebrum (scored 0–8, Min/Med/Ma) #**				
Control	0/0/0	0/0/0	2/2/3	0/0/0	2/2/3
BPC 157 10 μg/kg	0/0/0	0/0/0	0/1/1 *	0/0/0	0/1/1 *
BPC 157 10 ng/kg	0/0/0	0/0/0	0/1/1 *	0/0/0	0/1/1 *
	Neuronal damage in the karyopyknotic areas, %, Means ± SD (10 HPF, 400×)				
Control	0 ± 0	0 ± 0	50 ± 15	0 ± 0	50 ± 15
BPC 157 10 μg/kg	0 ± 0	0 ± 0	5 ± 5 *	0 ± 0	5 ± 5 *
BPC 157 10 ng/kg	0 ± 0	0 ± 0	5 ± 5 *	0 ± 0	5 ± 5 *
	Hemorrhage (% of total area)				
Control	25 ± 5	30 ± 5	25 ± 5	30 ± 5	25 ± 5
BPC 157 10 μg/kg	2 ± 2 *	5 ± 1 *	10 ± 5 *	10 ± 5 *	10 ± 5 *
BPC 157 10 ng/kg	2 ± 2 *	7 ± 1 *	12 ± 5 *	13 ± 5 *	11 ± 5 *
	Edema (scored 0–3, Min/Med/Max)				
Control	3/3/3	3/3/3	3/3/3	3/3/3	3/3/3
BPC 157 10 μg/kg	1/1/1 *	1/1/1 *	1/1/1 *	1/1/1 *	1/1/1 *
BPC 157 10 ng/kg	1/1/1 *	1/1/1 *	1/1/1 *	1/1/1 *	1/1/1 *
**Treatment**	**Cerebellum (scored 0–8, Min/Med/Max)**				
Control	0/0/0	0/0/0	2/2/3	0/0/0	2/2/3
BPC 157 10 μg/kg	0/0/0	0/0/0	0/1/1 *	0/0/0	0/1/1 *
BPC 157 10 ng/kg	0/0/0	0/0/0	0/1/1 *	0/0/0	0/1/1 *
	Neuronal damage in the karyopyknotic areas, %, Means ± SD (10 HPF, 400×)				
Control	0 ± 0	0 ± 0	40 ± 10	0 ± 0	40 ± 10
BPC 157 10 μg/kg	0 ± 0	0 ± 0	5 ± 5 *	0 ± 0	5 ± 5 *
BPC 157 10 ng/kg	0 ± 0	0 ± 0	5 ± 5 *	0 ± 0	5 ± 5 *
	Hemorrhage (% of total area)				
Control	0 ± 0	0 ± 0	0 ± 0	0 ± 0	0 ± 0
BPC 157 10 μg/kg	0 ± 0	0 ± 0	0 ± 0	0 ± 0	0 ± 0
BPC 157 10 ng/kg	0 ± 0	0 ± 0	0 ± 0	0 ± 0	0 ± 0
	Edema (scored 0–3, Min/Med/Max)				
Control	3/3/3	3/3/3	3/3/3	3/3/3	3/3/3
BPC 157 10 μg/kg	1/1/1 *	1/1/1 *	1/1/1 *	1/1/1 *	1/1/1 *
BPC 157 10 ng/kg	1/1/1 *	1/1/1 *	1/1/1 *	1/1/1 *	1/1/1 *
**Treatment**	**Hippocampus (scored 0–8, Min/Med/Max)**				
Control	0/0/0	0/0/0	2/2/2	0/0/0	2/2/2
BPC 157 10 μg/kg	0/0/0	0/0/0	0/1/1 *	0/0/0	0/1/1 *
BPC 157 10 ng/kg	0/0/0	0/0/0	0/1/1 *	0/0/0	0/1/1 *
	Neuronal damage in the karyopyknotic areas, %, Means ± SD				
Control	0 ± 0	0 ± 0	30 ± 10	0 ± 0	30 ± 10
BPC 157 10 μg/kg	0 ± 0	0 ± 0	5 ± 5 *	0 ± 0	5 ± 5 *
BPC 157 10 ng/kg	0 ± 0	0 ± 0	5 ± 5 *	0 ± 0	5 ± 5 *
	Hemorrhage (% of total area)				
Control	0 ± 0	10 ± 5	10 ± 5	10 ± 5	10 ± 5
BPC 157 10 μg/kg	0 ± 0	0 ± 0 *	0 ± 0 *	0 ± 0 *	0 ± 0 *
BPC 157 10 ng/kg	0 ± 0	0 ± 0 *	0 ± 0 *	0 ± 0 *	0 ± 0 *
	Edema (scored 0–3, Min/Med/Max)				
Control	2/2/2	3/3/3	3/3/3	3/3/3	3/3/3
BPC 157 10 μg/kg	1/1/1 *	1/1/1 *	1/1/1 *	1/1/1 *	1/1/1 *
BPC 157 10 ng/kg	1/1/1 *	1/1/1 *	1/1/1 *	1/1/1 *	1/1/1 *
**Treatment**	**Hypothalamus (scored 0–8, Min/Med/Max)**				
Control	0/0/0	0/0/0	2/2/2	0/0/0	2/2/2
BPC 157 10 μg/kg	0/0/0	0/0/0	0/1/1 *	0/0/0	0/1/1 *
BPC 157 10 ng/kg	0/0/0	0/0/0	0/1/1 *	0/0/0	0/1/1 *
	Neuronal damage in the karyopyknotic areas, %, Means ± SD				
Control	0 ± 0	0 ± 0	20 ± 5	0 ± 0	20 ± 5
BPC 157 10 μg/kg	0 ± 0	0 ± 0	5 ± 5 *	0 ± 0	5 ± 5 *
BPC 157 10 ng/kg	0 ± 0	0 ± 0	5 ± 5 *	0 ± 0	5 ± 5 *
	Hemorrhage (% of total area)				
Control	0 ± 0	10 ± 5	10 ± 5	10 ± 5	10 ± 5
BPC 157 10 μg/kg	0 ± 0	0 ± 0 *	0 ± 0 *	0 ± 0 *	0 ± 0 *
BPC 157 10 ng/kg	0 ± 0	0 ± 0 *	0 ± 0 *	0 ± 0 *	0 ± 0 *
	Edema (scored 0–3, Min/Med/Max)				
Control	2/2/2	3/3/3	3/3/3	3/3/3	3/3/3
BPC 157 10 μg/kg	1/1/1 *	1/1/1 *	1/1/1 *	1/1/1 *	1/1/1 *
BPC 157 10 ng/kg	1/1/1 *	1/1/1 *	1/1/1 *	1/1/1 *	1/1/1 *

# combined score (0–8)—semiquantitative neuropathological scoring system; sum of affected areas with infarction and karyopyknotic cells.

**Table 3:** Number of CD20-, CD3-, CD68-(PG-M1) and CD163-positive cells in the bile duct-ligated rats following ligation (time 0) at the sequential periods (application sacrifice) to illustrate the effect of both early treatment and delayed treatment following ligation. * *p* ˂ 0.05, at least, vs. control.

Regimens	Early Regimen			Delayed Regimen	
Periods	0–30 min	0–5 h	0–24 h	4.30 h–5 h	5 h–24 h
**Treatment**	**Protein CD20 (Means ± SD)**				
Control	1 ± 1	1 ± 0	36 ± 3	10 ± 2	34 ± 3
BPC 157 10 μg/kg	0 ± 0 *	1 ± 0	0 ± 0 *	1 ± 1 *	0 ± 0 *
BPC 157 10 ng/kg	0 ± 0 *	1 ± 0	0 ± 0 *	1 ± 1 *	0 ± 0 *
**Treatment**	**Protein CD3 (Means ± SD)**				
Control	2 ± 0	8 ± 1	21 ± 4	1 ± 0	23 ± 3
BPC 157 10 μg/kg	1 ± 0 *	0 ± 0 *	1 ± 0 *	0 ± 0 *	1 ± 0 *
BPC 157 10 ng/kg	1 ± 0 *	0 ± 0 *	1 ± 0 *	0 ± 0 *	1 ± 0 *
**Treatment**	**Protein CD68 (PG-M1) (Means ± SD)**				
Control	16 ± 2	24 ± 2	18 ± 2	20 ± 3	20 ± 2
BPC 157 10 μg/kg	0 ± 0 *	0 ± 0 *	0 ± 0 *	0 ± 0 *	0 ± 0 *
BPC 157 10 ng/kg	0 ± 0 *	0 ± 0 *	0 ± 0 *	0 ± 0 *	0 ± 0 *
	**Protein CD163 (Means ± SD)**				
Control	0 ± 0	8 ± 0	1 ± 0	7 ± 0	1 ± 0
BPC 157 10 μg/kg	0 ± 0	1 ± 0 *	0 ± 0 *	1 ± 0 *	0 ± 0 *
BPC 157 10 ng/kg	0 ± 0	1 ± 0 *	0 ± 0 *	1 ± 0 *	0 ± 0 *

## Data Availability

The data presented in this study are available on request from the corresponding author.
